# A conformation-specific ON-switch for controlling CAR T cells with an orally available drug

**DOI:** 10.1073/pnas.1911154117

**Published:** 2020-06-17

**Authors:** Charlotte U. Zajc, Markus Dobersberger, Irene Schaffner, Georg Mlynek, Dominic Pühringer, Benjamin Salzer, Kristina Djinović-Carugo, Peter Steinberger, Annika De Sousa Linhares, Nicole J. Yang, Christian Obinger, Wolfgang Holter, Michael W. Traxlmayr, Manfred Lehner

**Affiliations:** ^a^St. Anna Children's Cancer Research Institute (CCRI), 1090 Vienna, Austria;; ^b^Christian Doppler Laboratory for Next Generation CAR T Cells, 1090 Vienna, Austria;; ^c^BOKU Core Facility Biomolecular & Cellular Analysis, BOKU-University of Natural Resources and Life Sciences, 1190 Vienna, Austria;; ^d^Department of Structural and Computational Biology, Max Perutz Labs, University of Vienna, 1030 Vienna, Austria;; ^e^Department of Biochemistry, Faculty of Chemistry and Chemical Technology, University of Ljubljana, 1000 Ljubljana, Slovenia;; ^f^Institute of Immunology, Medical University of Vienna, 1090 Vienna, Austria;; ^g^Department of Immunology, Harvard Medical School, Boston, MA 02115;; ^h^Department of Chemistry, Institute of Biochemistry, BOKU-University of Natural Resources and Life Sciences, 1190 Vienna, Austria;; ^i^St. Anna Kinderspital, Department of Pediatrics, Medical University of Vienna, 1090 Vienna, Austria

**Keywords:** protein engineering, lipocalin, CAR T cell, CID, alternative scaffold

## Abstract

Molecular ON-switches in which a chemical compound induces protein–protein interactions can allow cellular function to be controlled with small molecules. ON-switches based on clinically applicable compounds and human proteins would greatly facilitate their therapeutic use. Here, we developed an ON-switch system in which the human retinol binding protein 4 (hRBP4) of the lipocalin family interacts with engineered hRBP4 binders in a small molecule-dependent manner. Two different protein scaffolds were engineered to bind to hRBP4 when loaded with the orally available small molecule A1120. The crystal structure of an assembled ON-switch shows that the engineered binder specifically recognizes the conformational changes induced by A1120 in two loop regions of hRBP4. We demonstrate that this conformation-specific ON-switch is highly dependent on the presence of A1120, as demonstrated by an ∼500-fold increase in affinity upon addition of the small molecule drug. Furthermore, the ON-switch successfully regulated the activity of primary human CAR T cells in vitro. We anticipate that lipocalin-based ON-switches have the potential to be broadly applied for the safe pharmacological control of cellular therapeutics.

## Significance

Molecular ON-switches are important tools in chemical biology, enabling protein–protein interactions to be regulated by small molecules. However, currently available ON-switches that induce conditional heterodimerization are suboptimal for therapeutic applications. In this study, we present an ON-switch system based on human retinol binding protein 4 (hRBP4) and the orally available small molecule A1120. Two distinct protein scaffolds, FN3 and rcSso7d, were successfully engineered to bind to hRBP4 in a small molecule-dependent manner, demonstrating the flexibility of the system. The binders specifically associated with the drug-induced conformation of hRBP4. Our study demonstrates that lipocalin-based ON-switches can enable specific regulation of protein heterodimerization and provides proof of concept for potential applications in controlling the activity of human CAR T cells.

The ability to control protein–protein interactions with small chemical compounds can open up exciting applications across various fields such as cell biology, immunology, and immunotherapy. These switchable systems are commonly known as chemically induced dimerization (CID) systems (1, 2). In general, in a CID the interaction between two proteins can be triggered by a small molecule, and therefore, CID systems can also be regarded as molecular ON-switches. The only molecular ON-switch that has been used in humans in vivo is based on a mutated version of FK506 binding protein (FKBP) 12, which is homodimerized upon administration of the small molecule AP1903 (3). However, for many applications it is necessary to regulate the interaction of two different proteins. Indeed, various systems have been introduced that enable such conditional heterodimerization (4–8), including the FRB/FKBP system that is used extensively in vitro. However, their clinical translation is limited due to unfavorable characteristics of the small molecule or the nonhuman origin of protein components (7, 9–12). Thus, an effective molecular ON-switch that can induce heterodimerization in a clinically relevant setting is still lacking.

One important application of ON-switches is the regulation of T cells that are genetically engineered to express chimeric antigen receptors (CARs). CARs consist of an extracellular antigen-binding moiety that is fused via a transmembrane region to an intracellular signaling domain derived from the T cell receptor complex and from costimulatory molecules (13, 14). Upon recognition of specific antigens on target cells, CARs trigger the release of cytokines and cytotoxic mediators. CAR T cells targeting the B cell marker CD19 have been impressively effective in the treatment of B cell malignancies such as acute lymphoblastic leukemia and lymphomas, recently gaining US Food and Drug Administration (FDA) approval (13). However, a significant limitation of this therapy is the inability to control CAR T cells after they are administered to the patient. This often leads to severe adverse events, such as neurological toxicities, organ dysfunction, and cytokine release syndrome (13, 15–17). Therefore, molecular tools which enable regulation of CAR T cell activity in vivo are urgently needed.

In this study, we aimed at generating a type of molecular ON-switch that matches two important design criteria: the usage of 1) an orally available small molecule with a favorable safety profile in vivo and 2) a human protein that undergoes a drug-induced conformational switch. We hypothesized that human lipocalins are ideally suited for such a molecular ON-switch. Lipocalins possess a β-barrel fold with an internal hydrophobic ligand-binding pocket, which can bind a range of different hydrophobic small molecules, depending on the shape and biochemical property of the binding pocket (18). Moreover, some lipocalins undergo conformational change upon binding to a small molecule (19–24). Thus, we hypothesized that other proteins could be engineered to specifically recognize the small molecule-loaded conformation of a lipocalin, forming the basis of a molecular ON-switch. In this proof-of-concept study, we used two different binder scaffolds: 1) reduced charge Sso7d (rcSso7d), which is a charge-neutralized version of a small (7 kDa), hyperthermostable protein derived from the archaeon *Sulfolobus solfataricus* (25, 26), and 2) the tenth type III domain of human fibronectin (FN3) with a molecular weight of 10 kDa (27–29).

Here, we demonstrate that lipocalin-based molecular ON-switches can be designed to be specifically regulated with an orally available small compound. We present ON-switches in which the affinity between the human lipocalin retinol binding protein 4 (hRBP4) and its engineered binders is increased up to 550-fold upon addition of the small molecule drug A1120. The crystal structure of the assembled ON-switch showed that the engineered binder specifically recognizes A1120-induced conformational changes in hRBP4. Finally, we show that this molecular ON-switch can be used to regulate cytotoxic activity and cytokine production of primary human CAR T cells, illustrating a potential future application of lipocalin-based ON-switches.

## Results

### Designing a Lipocalin-Based Molecular ON-Switch System.

**Fig. 1.**
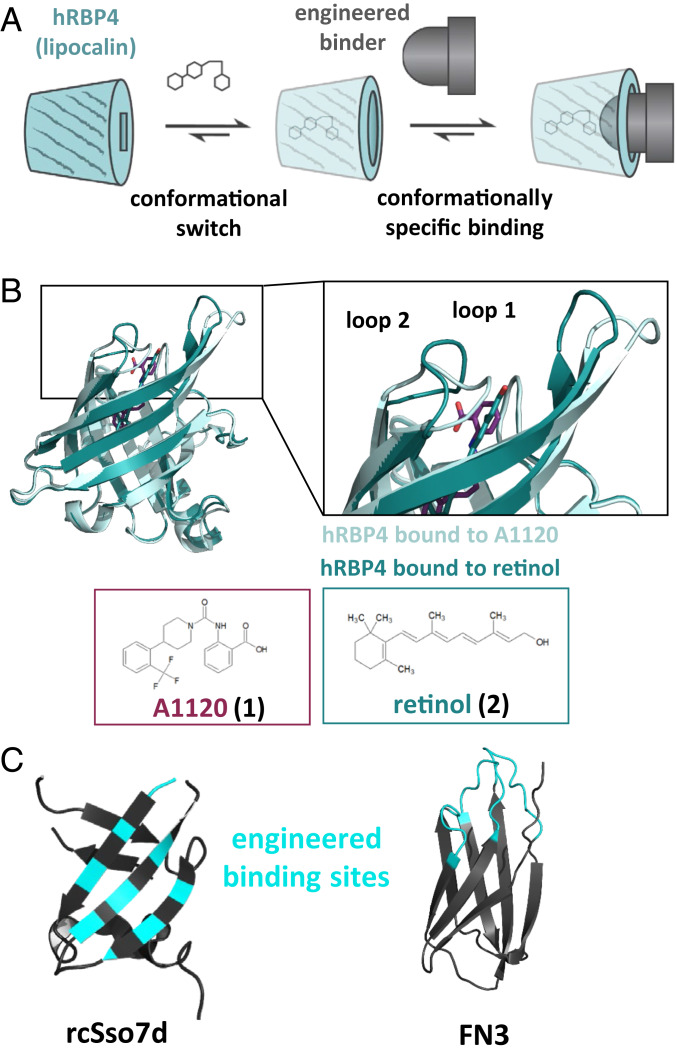
General principle of lipocalin-based ON-switches. (*A*) Schematic representation of molecular ON-switches based on a human lipocalin and an engineered binder scaffold, which heterodimerize upon addition of a small molecule. (*B*) Overlay of the crystal structures of the lipocalin hRBP4 bound to either retinol (dark green, PDB ID code 1RBP (30)) or A1120 (light green, PDB ID code 3FMZ (31)). The A1120-induced conformational switch in two loop regions of hRBP4 is depicted in the enlarged picture. The chemical structures of A1120 and retinol were generated with ChemSketch. (*C*) Binders derived from the rcSso7d (K-Ras-specific mutant depicted; PDB ID code 5UFQ (33)) or FN3 scaffold (PDB ID code 1TTG (34)) were engineered to recognize hRBP4 loaded with A1120. Randomized amino acid positions within the rcSso7d and FN3 library are colored in cyan. The figures in *B* and *C* were generated using the PyMOL Molecular Graphics System (version 1.3, Schrödinger, LLC).

In this study, we aimed at engineering binder scaffolds to specifically recognize a lipocalin in the presence of a small compound. The resulting small molecule-induced protein–protein interaction can be described as a molecular ON-switch (Fig. 1*A*). To test whether efficient ON-switches can be engineered based on human lipocalins, we searched the literature for lipocalins that undergo conformational changes upon ligand binding. The most promising candidate was human retinol binding protein 4 (hRBP4), which transports retinol in plasma (18, 30). This human protein provides several substantial advantages: It does not contain any *N*-glycosylation sites or free cysteines and has been described as a stable, well-expressed, and monomeric protein (18). In 2009, Motani et al. introduced the synthetic hRBP4-specific drug A1120 that triggers dissociation of hRBP4 from its carrier protein transthyretin (TTR). This, in turn, results in reduced plasma levels of hRBP4, which—at that time—were believed to counteract insulin resistance (31). Importantly, upon binding of A1120, a conformational switch in two loop regions at the entrance of the binding pocket has been observed (31, 32) (Fig. 1*B*). Therefore, we hypothesized that protein binder scaffolds can be engineered to specifically recognize the A1120-induced conformation of hRBP4 with high affinity while only weakly interacting with hRBP4 in the absence of A1120.

To test this hypothesis, we chose the two binder scaffolds rcSso7d (25) and FN3 (35) for yeast display selection experiments. Whereas the engineered binding surface of rcSso7d is composed of rigid β-strands, that of FN3 domains is located on flexible loop regions (Fig. 1*C*). The choice of these completely different types of binder scaffolds enabled us to test whether it is possible to generate hRBP4-based ON-switches independent of the structure of the engineered interacting binding surface.

### Generation and Characterization of hRBP4-Based ON-Switches.

**Fig. 2.**
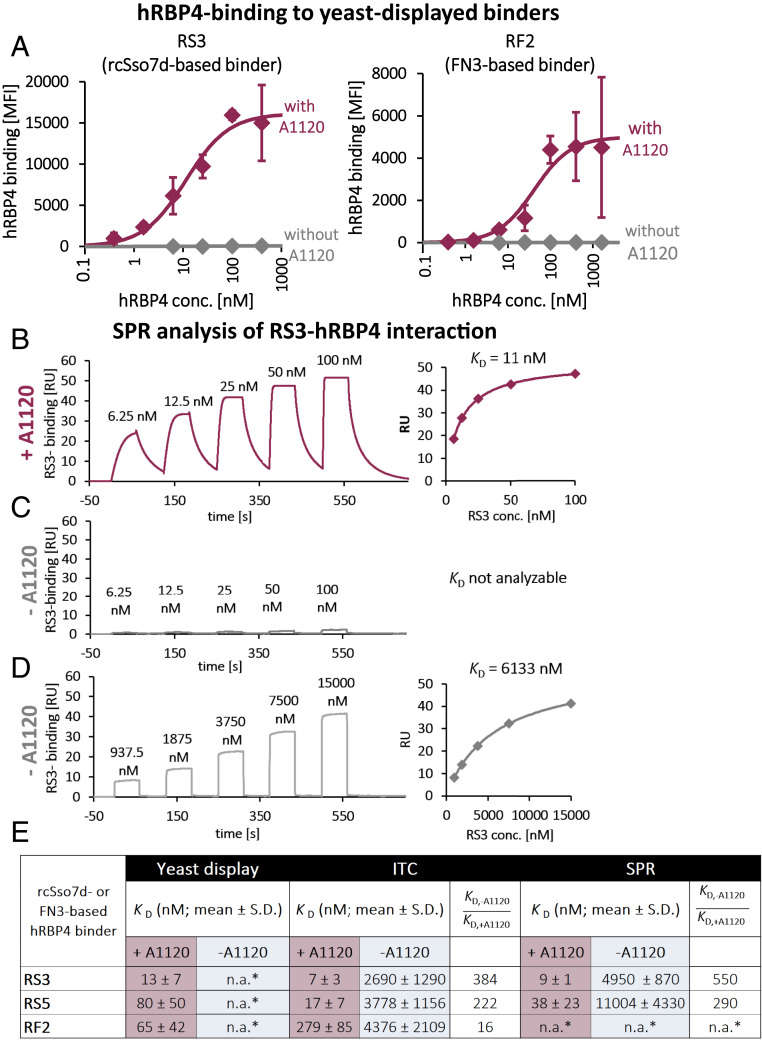
Selected rcSso7d- and FN3-based binders specifically recognize the A1120-induced conformation of hRBP4. (*A*) Enriched binders were displayed on the surface of yeast, followed by titration of hRBP4, both in the presence (5 µM) and absence of A1120, respectively. Subsequently, binding intensity was analyzed by flow cytometry. Averages of median fluorescence intensities (MFIs) ± SDs of three independent experiments are shown. Data were fitted to a 1:1 binding model (solid lines) to calculate the *K*_D_ values shown in *E*. (*B*) Single-cycle kinetics (SCK) SPR experiment with hRBP4 immobilized on a sensor chip and titrated with RS3 in the presence of 5 µM A1120. *K*_D_ values were calculated by steady-state analysis (diagram on the *Right*). (*C*) SCK experiment in the absence of A1120 with the same RS3 concentrations used in *B*. (*D*) SCK experiment in the absence of A1120 with higher RS3 concentrations. *K*_D_ values were calculated by steady-state analysis (diagram on the *Right*). Representative diagrams and *K*_D_ values of three (*C*) or four (*B* and *D*) independent experiments are shown. (*E*) Overview of *K*_D_ values of screened binders as determined by flow cytometric analysis (*n* = 3), ITC (*n* = 4), or SPR (*n* = 4) (*n.a., not analyzable).

We used randomly mutated rcSso7d and FN3 libraries (25, 35) and selected those yeast-displayed libraries for binding to hRBP4 in the presence of A1120. To yield 99% saturation of hRBP4, the A1120 concentration needs to be ∼100-fold above the dissociation constant (*K*_D_) value. Therefore, to achieve virtually full saturation of hRBP4, a concentration of 5 µM A1120 was chosen throughout this study unless indicated otherwise, which is ∼600-fold above the *K*_D_ reported in the literature (31). To improve the specificity of the binders for the A1120-induced conformation of hRBP4, one round of negative selection was included, in which the libraries were selected for nonbinding in the absence of A1120 (*SI Appendix*, Fig. S1*A*). After several rounds of selection, including two rounds of affinity maturation by using error-prone PCR, enriched clones were sequenced (sequences are shown in *SI Appendix*, Fig. S1*B*). The most frequent binders were termed according to their target (hRBP4) and origin (rcSso7d or FN3). Thus, rcSso7d-based binders are termed RS1 to RS5, and FN3-based mutants are called RF1 to RF3. Subsequently, those enriched binders were displayed on yeast individually and analyzed for binding to hRBP4 in the presence (5 µM) and absence of A1120. Remarkably, all tested binders based on both rcSso7d and FN3 recognized hRBP4 only in the A1120-loaded conformation (Fig. 2*A* and *SI Appendix*, Fig. S1*C*). Thus, these hRBP4-based systems represent molecular ON-switches that can be turned on by administration of A1120. In the “on state,” the affinities cover virtually the full nanomolar range depending on the binder that is used, with the highest affinity being 2 nM for RS1 (*SI Appendix*, Fig. S1*E*).

**Fig. 3.**
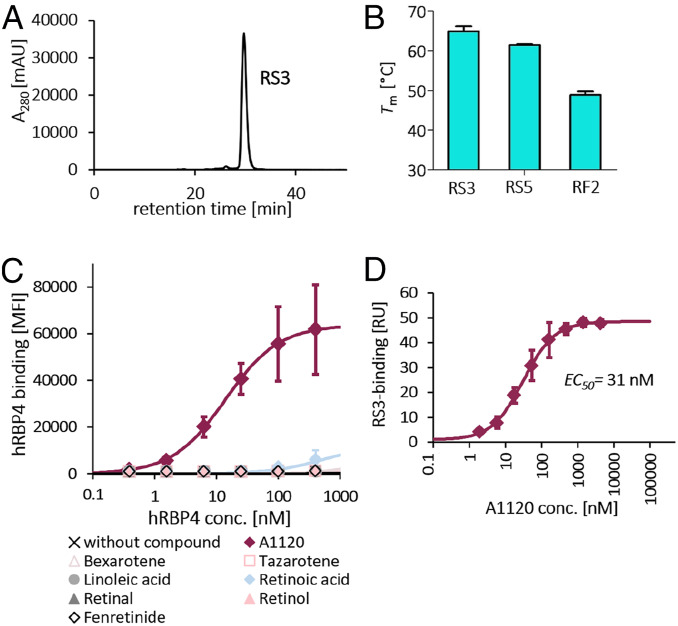
Detailed biochemical analysis of enriched binders and of the hRBP4-RS3 ON-switch. (*A*) SEC profile of one selected rcSso7d-based hRBP4 binder (RS3). One representative measurement of three independent experiments is shown. (*B*) *T*_m_ values of selected RS and RF binders as determined by DSC (mean ± SD of four independent experiments). (*C*) The binder RS3 was displayed on the surface of yeast, followed by titration of soluble hRBP4 either in the absence or in the presence (5 µM) of different known hRBP4 ligands. hRBP4 binding was measured by flow cytometry (mean ± SD of three independent experiments). (*D*) hRBP4 was immobilized on an SPR chip, and the interaction with a fixed concentration of RS3 (100 nM) was analyzed at different A1120 concentrations. Steady-state RS3 binding levels of three independent experiments were plotted against A1120 concentration, followed by fitting the resulting curve to a three-parameter model by nonlinear regression.

Based on the binding data (*SI Appendix*, Fig. S1 *C* and *E*) and expression levels (*SI Appendix*, Fig. S1*D*), three mutants of each scaffold were expressed solubly and analyzed for their tendency to aggregate by size exclusion chromatography (SEC) (Fig. 3*A* and *SI Appendix*, Fig. S2). While RS1, RF1, and RF3 showed strong aggregation, the mutants RS3, RS5, and RF2 eluted as single peaks, with only minor aggregation observed for RF2 (Fig. 3*A* and *SI Appendix*, Fig. S2). These three nonaggregating mutants were further analyzed by differential scanning calorimetry (DSC), showing that the rcSso7d-based binders were considerably more stable than the FN3-based mutant, with melting temperature (*T*_m_) values of 65.1 °C ± 1.0 °C and 61.7 °C ± 0.5 °C for RS3 and RS5, respectively, compared with 48.8 °C ± 0.7 °C for RF2 (Fig. 3*B*).

Since the affinities between the engineered binders and hRBP4 in the absence of A1120 are too weak to be measured by titration on the surface of yeast (Fig. 2*A* and *SI Appendix*, Fig. S1*C*), it was not possible to determine the affinity ratio in the presence vs. absence of A1120 using this approach. Therefore, the interactions between the three most promising engineered binders and hRBP4 were additionally analyzed by isothermal titration calorimetry (ITC) (*SI Appendix*, Fig. S3), yielding two important findings: First, the *K*_D_ values in the presence of A1120 closely match those determined by titrations on the surface of yeast (Fig. 2*E*). Second, the presence of A1120 strongly increases the affinity between hRBP4 and both rcSso7d-based binders (384-fold and 222-fold for RS3 and RS5, respectively; Fig. 2*E*), demonstrating that these ON-switches are highly specific for the A1120-loaded conformation of hRBP4. Although the influence of A1120 on the affinity of the FN3-based binder RF2 (16-fold, Fig. 2*E*) was not as pronounced as the affinity increases observed with rcSso7d-based binders, this still confirms that hRBP4-based ON-switches can be constructed using binder scaffolds with completely different binding surfaces.

Finally, the A1120-dependent interactions between rcSso7d-based binders and hRBP4 were further analyzed by surface plasmon resonance (SPR). Whereas analysis of the hRBP4-RS3 interaction in the presence of A1120 yielded a strong signal (Fig. 2*B*), in the absence of A1120 binding was hardly detectable (Fig. 2*C*), similar to the observations in the yeast surface titration experiments. Therefore, to be able to determine *K*_D_ values in the absence of A1120, SPR experiments were additionally performed with elevated RS3 concentrations, yielding a *K*_D_ of 6 µM in the experiment shown in Fig. 2*D* (average *K*_D_ values are shown in Fig. 2*E*). Similar A1120-dependent effects were observed with RS5 (*K*_D_ values depicted in Fig. 2*E*). Overall, for both RS3 and RS5 the *K*_D_ values obtained from SPR experiments closely match those derived from yeast surface titrations and ITC experiments (Fig. 2*E*).

Together, the ability of the engineered binders to specifically recognize the A1120-induced conformation of hRBP4 was analyzed with three different methods, yielding highly comparable results (Fig. 2*E*). For all further experiments the binder RS3 was chosen as the most promising candidate because it combines high affinity to A1120-loaded hRBP4 with high expression levels on the surface of yeast, monomeric behavior in SEC analysis, and high thermal stability. Most importantly, the affinity between RS3 and hRBP4 is increased upon addition of A1120 by several hundredfold (550-fold and 384-fold when analyzed by SPR and ITC, respectively).

### The Generated hRBP4-RS3 ON-Switch Is Highly Specific for A1120 and Tunable.

Another important prerequisite for broadly applicable molecular ON-switches is their orthogonality, i.e., their independence of other described small molecule ligands. Therefore, it is important to investigate whether the ON-switch based on hRBP4 and RS3 is solely turned on by A1120 or if other small molecules known to bind to hRBP4 also activate the hRBP4-RS3 interaction. For this purpose, RS3 was displayed on the surface of yeast and tested for binding to hRBP4 in the presence or absence of different natural and synthetic small molecule ligands of hRBP4. Again, strong binding was observed in the presence of A1120. Remarkably, in the presence of any other small molecule, binding of hRBP4 to RS3 was either not detectable at all or yielded only low signal at high hRBP4 concentrations (Fig. 3*C*). These data show that the ON-switch is highly specific for the A1120-induced conformation and is not triggered by other known hRBP4 ligands.

Next, we analyzed the dependency of the hRBP4-RS3 interaction on the A1120 concentration. For that purpose, hRBP4 was immobilized on an SPR chip, and binding of RS3 was analyzed at a fixed RS3 concentration. As expected, RS3 binding increased in an A1120 concentration–dependent manner, yielding an effective concentration (EC_50_) of 31 nM (Fig. 3*D*). This demonstrates that the activity of this molecular ON-switch can be fine-tuned when A1120 is administered at low nanomolar concentrations, especially in applications where the A1120 concentration can be precisely controlled.

### Structural Analysis Confirms Conformational Specificity of RS3 for A1120-Loaded hRBP4.

**Fig. 4.**
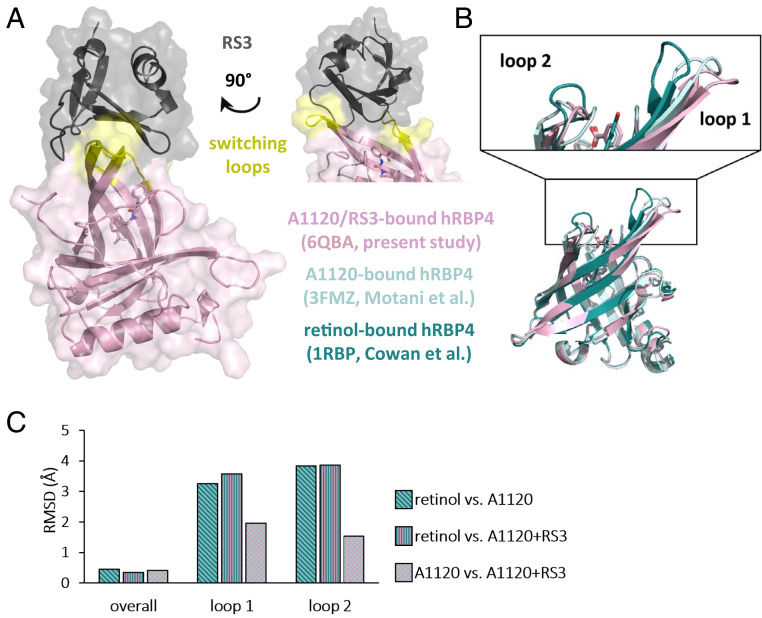
Structure of the hRBP4-RS3 ON-switch complex in the presence of A1120. (*A*) Crystal structure of the ON-switch complex at 1.8 Å resolution. Surface and cartoon representation of hRBP4 (pink) and RS3 (black). The switching loops of hRBP4 are indicated in yellow. On the *Right* the top part of the structure is shown after rotation by 90° around the vertical axis. (*B*) Overlay of different crystal structures of hRBP4 bound to either retinol (dark green, PDB ID code 1RBP (30)), A1120 (light green, PDB ID code 3FMZ (31)), or A1120 and RS3 (pink, PDB ID code 6QBA; RS3 is not depicted). The close-up view shows the switching loop regions of hRBP4 bound to either retinol (dark green) or A1120 (light green and pink). (*C*) Calculated RMSD values between the three represented hRBP4 crystal structures as indicated. All figures were generated using the PyMOL Molecular Graphics System (version 1.3, Schrödinger, LLC).

To investigate the molecular mechanism of the ON-switch, the crystal structure of the hRBP4-A1120-RS3 complex was solved at 1.8-Å resolution (data collection and refinement statistics are summarized in *SI Appendix*, Table S1) (36). X-ray crystallography revealed that RS3 binds to hRBP4 at the entrance of the ligand-binding pocket. More precisely, the engineered binder interacts with the two loop regions (shown in yellow in Fig. 4*A*) that have been described to be switched upon binding of A1120 (31, 32), confirming our hypothesis that these A1120-induced conformational changes enable the construction of a drug-dependent ON-switch.

To analyze the conformational switch in hRBP4 in more detail, the overlay of the crystal structures of hRBP4 bound to either retinol (30), A1120 (31), or A1120+RS3 is shown in Fig. 4*B* and RMSDs between Cα atoms of the three structures are presented in Fig. 4*C*. While the overall conformations of all three hRBP4 structures are highly similar, the abovementioned loop regions show substantial structural changes. Of note, in both loop 1 and loop 2 the RMSDs between retinol-bound hRBP4 and either of the two A1120-loaded structures (with or without RS3) were about twofold higher compared with the RMSDs in the A1120- vs. A1120+RS3-bound state (Fig. 4*C*). That is, those loop regions adopt similar conformations in the two A1120-loaded structures but are structurally distinct from the retinol-bound state. The observation that the presence of RS3 induces minor structural deviations in those loops of around 1.5 to 2 Å (A1120-bound vs. A1120+RS3-bound hRBP4) is not surprising since these loops also interact with the engineered binder. Together, these structural data confirm that RS3 specifically recognizes the A1120-induced conformational switch located in two loop regions at the entrance to the ligand-binding pocket of hRBP4.

### The Engineered hRBP4-Based ON-switch Enables the Control of CAR T Cell Activity.

**Fig. 5.**
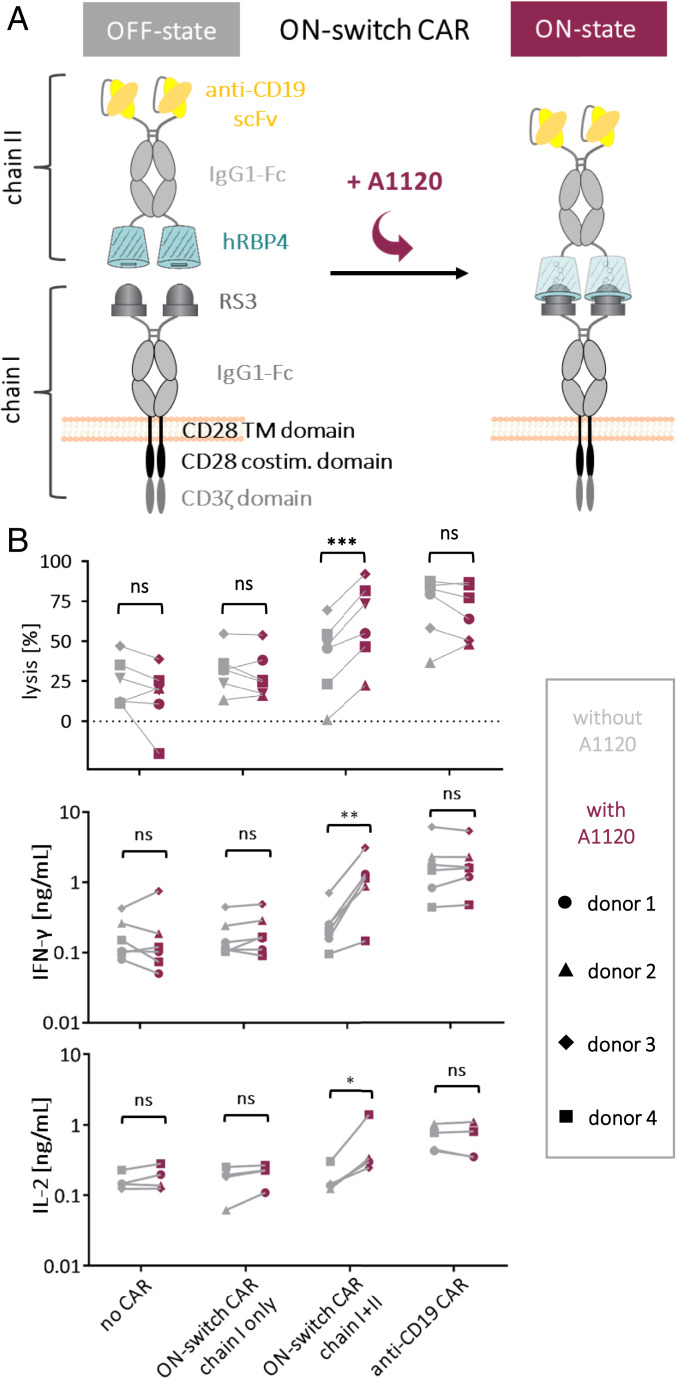
Incorporation of the ON-switch into a CAR. (*A*) Schematic mechanism of the ON-switch CAR. (*B*) Activation of primary human T cells expressing a CAR with an integrated ON-switch. Primary human T cells were electroporated with mRNA encoding either chain I only or both chains I and II of the ON-switch CAR or an anti-CD19 control CAR. As a negative control, T cells electroporated without any mRNA were included. Lysis of NALM6 target cells in the presence (5 µM) or absence of A1120 was determined by a luciferase-based cytotoxicity assay after 4 h of coculture with CAR T cells (effector:target ratio of 2:1). Target cells were blocked with 5% human serum for 15 min at 4 °C before effector cells were added. Statistical significance was calculated with GraphPad using the paired two-tailed Student’s *t* test. Supernatants of the cocultures were analyzed for secretion of the T cell–derived cytokines IFN-γ and IL-2. Statistical significance was calculated with GraphPad using the ratio paired *t* test. Data from four or six independent experiments with primary T cells from four different donors are shown. ns, not significant. ***P < 0.001, **P < 0.01, *P < 0.05.

Next, we applied our ON-switch for regulation of cellular therapy. In particular, regulation of CAR activity is still a big challenge in the field of CAR T cell therapy. For this application, an ON-switch based on an orally available drug such as A1120 is highly desired. We tested whether our engineered molecular switch can be used to control CAR function by constructing an ON-switch CAR that is composed of two chains. For proof of principle, we chose a design in which chain I contains the RS3 binder on a second-generation CAR backbone with an extracellular IgG1-Fc spacer domain, whereas chain II is composed of hRBP4 and an scFv directed against the B cell marker CD19, again connected by an IgG1-Fc spacer (Fig. 5*A* and *SI Appendix*, Fig. S5*A*). Importantly, chain II does not contain any transmembrane domain, which means that this soluble protein needs to be captured by chain I in the secretory pathway and/or on the cell surface. Thus, a functional CAR that contains both the antigen-binding scFv (anti-CD19) and the signaling domains is assembled only upon binding of RS3 to hRBP4, which can be controlled by addition of the small molecule A1120. On the other hand, if A1120 is not present, chain II cannot associate with chain I, thereby preventing activation of the CAR T cells (Fig. 5*A*).

Primary human T cells were electroporated with separate messenger RNAs (mRNAs) encoding the two chains of the ON-switch CAR. Chain I was detected on the T cell surface at high levels comparable with those of a CD19-specific control CAR (*SI Appendix*, Fig. S5*B*), which is composed of a single polypeptide chain and therefore independent of A1120 (*SI Appendix*, Fig. S5*A*). This shows that CARs containing an rcSso7d-based binder can be efficiently expressed by primary T cells (*SI Appendix*, Fig. S5*B*). Moreover, chain II bound to chain I was also detected, albeit at lower levels. Importantly, the chain II signal was dependent on the presence of A1120 (*SI Appendix*, Fig. S5*C*), demonstrating that the two components are able to assemble into the ON-switch CAR in an A1120-dependent manner.

Based on these preliminary results, we tested the functionality of the ON-switch CAR in primary human T cells in a cytotoxicity assay with CD19-positive NALM6 tumor cells. CAR T cells only expressing chain I showed cytotoxicity similar to T cells not expressing any CAR, both in the presence and absence of A1120 (Fig. 5*B*, *Top*), confirming that chain I by itself does not trigger T cell activation. On the other hand, the anti-CD19 control CAR efficiently lysed NALM6 target cells independently of A1120, as expected. Remarkably, T cells expressing both chains of the ON-switch CAR could be turned on by addition of A1120 (Fig. 5*B*, *Top*). More specifically, in the absence of A1120, cytotoxicity of ON-switch CAR T cells was similar to the negative control T cells expressing either no CAR or chain I only. In contrast, in the presence of A1120, lysis reached levels that were comparable to those achieved with the anti-CD19 control CAR. Similar effects were observed when the supernatants of these CAR T cell/NALM6 cocultures were analyzed for the T cell–derived cytokines IFN-γ and IL-2. In agreement with the cytotoxicity data, only in the presence of both chains of the ON-switch CAR were the cytokine levels significantly increased upon addition of A1120 (Fig. 5*B*, *Bottom*). Again, the levels that were reached with A1120 and the fully assembled ON-switch CAR were comparable to those of the anti-CD19 control CAR, demonstrating strong activation of T cell effector functions.

It should be noted that the differences in background activation of the T cells between different experiments can be explained by donor-specific variations. More specifically, the percentage of CD8^+^ T cells correlated with background lysis, even in the absence of any CAR expression (*SI Appendix*, Fig. S5*D*). Nevertheless, despite these assay-specific variations, these data clearly demonstrate that the activity of these CAR T cells can be switched on by administration of the orally available small molecule A1120.

To confirm that the A1120-regulated ON-switch CAR is antigen specific, similar experiments were additionally performed with Jurkat target cells transfected with CD19, as well as with their CD19-negative counterparts. As expected, only the CD19-positive target cells were killed by the ON-switch CAR T cells upon addition of A1120 (*SI Appendix*, Fig. S5 *E* and *F*).

**Fig. 6.**
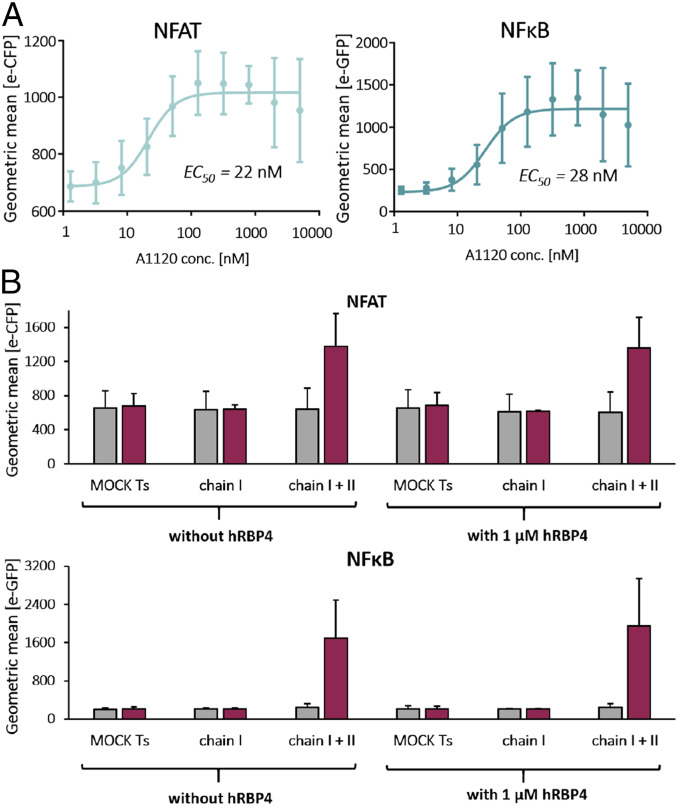
Influence of A1120 concentration and soluble hRBP4 on the ON-switch CAR function. Reporter Jurkat cells were electroporated with mRNA encoding both chains I and II of the ON-switch CAR and cocultured with NALM6 target cells (E:T = 1:2) for 20 h. Target cells were blocked with 10% human serum and 10% human IgG for 15 min at 4 °C before effector cells were added. Expression of the fluorescent reporter proteins was analyzed by flow cytometry. In *A* different concentrations of A1120 were administered to the cocultures, and EC_50_ values were calculated by fitting the data with a nonlinear regression model with a variable slope using GraphPad. In *B* the cells were incubated either without compound or with 5 µM A1120 and with or without 1 µM soluble hRBP4. Data shown in *A* and *B* are averages ± SDs of three independent experiments.

To precisely analyze the dependency of the ON-switch CAR activity on the A1120 concentration, we employed the well-defined reporter Jurkat T cell line, in which T cell activation is reported by expression of two different fluorescent proteins under the control of the transcription factors NFAT and NFκB, respectively. These dual reporter Jurkat cells were electroporated with mRNA encoding chains I and II of the ON-switch CAR, followed by cocultivation with NALM6 target cells. Titration of A1120 yielded EC_50_ values of 22 and 28 nM for NFAT and NFκB activation, respectively (Fig. 6*A* and *SI Appendix*, Fig. S6*B*; CAR expression is shown in *SI Appendix*, Fig. S6*A*), which are remarkably comparable to the EC_50_ obtained in the biochemical SPR binding assay (Fig. 3*D*; 31 nM). Thus, the potency of A1120 to turn on the hRBP4-based molecular ON-switch closely matches its ability to trigger T cell signaling in a CAR molecule. Similar to the assays with primary human T cells, A1120 did not induce reporter activation in Jurkat cells expressing either no CAR, chain I only, or the reference anti-CD19 CAR, again excluding any direct effect of A1120 on T cell activity (*SI Appendix*, Fig. S6*C*).

Finally, to test whether endogenous serum hRBP4 influences the function of the ON-switch CAR, we conducted dual-reporter Jurkat assays in the absence or presence of 1 µM hRBP4, which corresponds to the reported human plasma concentration (37, 38). More specifically, we investigated whether soluble hRBP4 1) blocks the assembly of chains I and II or 2) constitutively activates chain I expressing CAR T cells by binding to RS3. NFAT and NFκB signaling in ON-switch CAR T cells was highly dependent on A1120 and not reduced in the presence of soluble hRBP4, indicating that the added soluble RBP4 could not compete for the assembly of chains I and II (Fig. 6*B*). Moreover, soluble hRBP4 did not activate chain I expressing CAR T cells, either in the presence or in the absence of A1120 (Fig. 6*B*). Together, these experiments strongly suggest that endogenous hRBP4 does not cause blockade or uncontrolled activation of ON-switch CAR T cells.

## Discussion

In this study we generated a molecular ON-switch system, in which the interaction between a human lipocalin and an engineered binder scaffold can be controlled with an orally available small molecule. Molecular ON-switches for conditional heterodimerization are currently limited with regard to in vivo applicability, lack of orthogonality, and/or potential toxicities (4, 9–11). For example, the FRB/FKBP system can be regulated by rapamycin. However, due to its immunosuppressive activity, rapamycin is considered to be suboptimal. A very recent preclinical CAR study showed promising results with lower concentrations of this drug (39). Nevertheless, if available, a safe compound without any immunocompromising effect—like A1120—would be preferred, especially for immunotherapeutic applications. An alternative to rapamycin is the usage of its derivatives (so-called rapalogs), such as AP21967, which also have several drawbacks. Apart from residual immunosuppressive activity, the synthesis of rapalogs is difficult and cost intensive, and potential contamination with rapamycin is a risk potentially resulting in enhanced immunosuppression. Therefore, rapalogs are considered to be suboptimal for broad clinical application (9–12, 39).

In contrast, the small molecule A1120 was originally developed for long-term treatment of insulin resistance and was later also tested for treatment of age-related macular degeneration (AMD) (31, 40). hRBP4 is the transport molecule for retinol in human plasma. Due to its relatively small size (21 kDa), hRBP4 would be rapidly cleared from the circulation by the kidneys. This is prevented by complexation with another plasma protein called transthyretin (TTR) (41, 42). A1120 was developed for blocking this interaction of hRBP4 with TTR (31, 32), resulting in increased filtration of hRBP4 through the kidneys. This is the mechanistic basis for the original applications of A1120 mentioned above, where the overall goal was a reduction of hRBP4 and/or retinoid levels in the plasma. Importantly, three different research groups have shown that oral administration of A1120 to mice does not cause any systemic toxicities, even at high doses up to 30 mg/kg and for up to 5 mo (31, 32, 40, 43). Although the free plasma concentration of A1120 has not been reported in the literature, the data of Du et al. suggest that low micromolar serum RBP4 levels could be virtually saturated with A1120 (44). There is thus substantial evidence for efficient loading of hRBP4 with this drug in vivo, which is the critical parameter for the function of such an ON-switch. Note that A1120 is approved by the FDA for testing in patients as an investigational drug, and clinical trials for long-term treatment of AMD and inherited Stargardt macular dystrophy are under development (40, 45).

Another recently introduced ON-switch is based on the human protein BCL-xL and the small molecule ABT-737 (4). In that system, antibody fragments (Fabs) were successfully engineered to bind to a newly generated epitope consisting of both BCL-xL and the solvent-exposed portion of ABT-737. These Fabs bind to BCL-xL with high affinity only in the presence of ABT-737. However, since ABT-737 blocks the antiapoptotic function of BCL-xL and other Bcl-2 family members, administration of ABT-737 is associated with platelet and lymphocyte toxicities (46). Moreover, ABT-737 is not orally available (47), as is often the case with small molecules with a molecular weight > 500 Da. Finally, since a large portion of ABT-737 contributes to the epitope, this small molecule is also recognized by the Fab when bound to the homologous protein BCL-W, albeit with lower affinity. This suggests that using solvent-exposed small molecules, which form part of the recognized epitope, may limit the achievable specificity of the resulting ON-switches.

In contrast, the small molecule A1120 used in our lipocalin-based ON-switch is almost completely hidden in the ligand-binding pocket of hRBP4 (*SI Appendix*, Fig. S7*B*). For that reason, it could have been anticipated that this precludes efficient discrimination between the ligand-bound vs. the unbound state of hRBP4. Remarkably, the binders recognized hRBP4 with up to 550-fold higher affinity in the presence vs. absence of A1120. More broadly, this demonstrates that in molecular ON-switches the small molecule does not need to be solvent exposed for efficient switching behavior, provided that a conformational switch is triggered in the protein which enables allosteric recognition. We confirmed this hypothesis by analyzing the hRBP4-A1120-RS3 complex by X-ray crystallography and showing that RS3 indeed mostly interacts with the two loops of hRBP4 (36), which have been reported to change their conformation upon binding of A1120 (31, 32). Notably, comparison of those switching loop regions in the retinol-bound vs. either of the two A1120-bound structures revealed RMSD values of around 3.3 to 3.9 Å, despite only minor differences (∼0.4 Å) between the overall structures (Fig. 4*C*). For comparison, RMSDs between two protein cores with only 20% sequence identity are typically in the range of 2 Å (48), indicating that the A1120-induced structural deviations in those loop regions are substantial, potentially explaining the high efficiency of the ON-switch. Furthermore, an overlay of the RS3-bound hRBP4/A1120 complex with the retinol-bound hRBP4 structure shows that in the retinol-bound state loop 2 of hRBP4 would sterically clash with residues 23 and 25 of RS3 (*SI Appendix*, Fig. S4), further supporting the hypothesis that the binder recognizes the A1120-induced conformation of hRBP4. Importantly, this allosteric mechanism automatically avoids direct recognition of the small molecule and therefore off-target interactions with other proteins bound to the same compound, limiting unwanted side effects.

The fact that the engineered binders recognize a specific conformational state of hRBP4 bears the risk that other small molecules binding to hRBP4 may also trigger the ON-switch system. However, we demonstrated that the interaction of RS3 with hRBP4 bound to other known natural and synthetic ligands is almost undetectable. This confirms that lipocalin-based ON-switches can be designed to be specifically activated by a given small molecule but not by other small molecules, even if they bind to the hydrophobic pocket of the lipocalin. Together, these data strongly suggest that our ON-switch is orthogonal, i.e., largely independent of other small molecules.

One important application of molecular ON-switches is the regulation of CAR T cell activity for cancer immunotherapy. ON-switches based on human proteins and an orally available drug would facilitate the regulation of CAR T cell function in vivo. We demonstrated that the hRBP4-A1120-RS3-based ON-switch can turn on primary human CAR T cells using A1120 in vitro. This was achieved by splitting the CAR into two polypeptide chains, which only assemble upon interaction of hRBP4 and RS3. The chosen design could be considered a challenge as both chain I and chain II are expressed by the T cell. This means that chain II (which is a soluble protein) needs to be captured on the surface—or in the endoplasmic reticulum (ER) or Golgi during secretion—in order to promote assembly of the CAR. Alternatively, the soluble protein might accumulate locally and opsonize the target cells before being captured by the CAR T cells. Nevertheless, the activation levels achieved with the ON-switch CAR were comparable to those of an anti-CD19 control CAR.

Our CAR assembly system is different from that recently used by Lim and colleagues, where both chains were expressed in a membrane-anchored version (10) (*SI Appendix*, Fig. S7*A*). While the strategy with two membrane-anchored constructs prevents loss of any soluble CAR chain due to diffusion, it harbors the disadvantage that the interaction with the target cells is not regulated. Instead, only the activation of CAR signaling, and not binding to the antigen, can be controlled with the small molecule. This might potentially result in CAR T cells becoming trapped in antigen-positive tissues despite the absence of the small molecule. In our CAR system, however, both CAR signaling and the physical interaction of the CAR T cells with the target cells are prevented in the absence of A1120, which provides an additional layer of safety compared with just turning off CAR signaling. Apart from regulation of CAR T cells based on protein switches, alternative approaches were recently introduced, such as suppression of TCR and CAR signaling by dasatinib (49–51). Despite not being specific for CAR T cells, the application of this kinase inhibitor represents an elegant alternative approach for controlling CAR T cells in vivo.

Summing up, we introduced an ON-switch system based on a human lipocalin, an orally available drug, and two different engineered binder scaffolds. Notably, whereas the engineered binding sites on rcSso7d-based binders are located on a rigid β-sheet (25), the binding surfaces on FN3-based binders are composed of flexible loop regions (27, 28) (Fig. 1*C*). While ON-switches based on the human scaffold FN3 could be engineered successfully, those based on the nonhuman scaffold rcSso7d showed even higher dependency on A1120. Therefore, in this first proof-of-concept study we focused on the nonhuman and potentially immunogenic scaffold rcSso7d. However, our findings that completely different binding sites can be engineered to specifically recognize small molecule-induced conformational changes in a lipocalin illustrate the flexibility of the system with regard to the choice of the engineered interaction partner. Consequently, we anticipate that other human binder scaffolds such as scFvs or fynomers (52) can also be used to construct hRBP4-based ON-switches. In addition, since small molecule-induced conformational changes have also been described for other lipocalins (19, 20, 22–24, 53), we expect that this type of molecular ON-switch is not limited to hRBP4 either. Finally, it has been observed both in nature (18, 54) and during protein engineering experiments in the laboratory (55, 56) that the lipocalin structure is highly tolerant to mutations, enabling adaptation for binding to different small molecular compounds. Thus, we anticipate that lipocalin-based ON-switches are flexible with regard to the choice of all three components: the lipocalin, the binder scaffold, and the regulating small molecule.

## Materials and Methods

A detailed description of all materials, equipment, and methods used in this study can be found in the *SI Appendix*.

### Isothermal Titration Calorimetry.

ITC experiments were conducted using a PEAQ Isothermal Titration Calorimeter Automated (Malvern Panalytical). rcSso7d- and FN3-based binders were dialyzed against the same buffer (phosphate-buffered saline (PBS), pH 7.4) as hRBP4. Ten micromolar hRBP4 was applied to the sample cell, and 100 µM of the respective binder were titrated in the presence (50 µM) or absence of A1120 with 1 µL injection volumes. In the case of ITC experiments in the presence of A1120, the solutions of both hRBP4 and the binder contained 50 µM A1120 to avoid buffer mismatch. Data analysis was performed with the PEAQ-ITC analysis software (Malvern Panalytical).

### Surface Plasmon Resonance.

SPR experiments were performed with the BiacoreT200 instrument (GE Healthcare). hRBP4 was covalently immobilized on a CM5 chip (GE Healthcare) using the method of amine coupling according to the manufacturer’s protocol (GE Healthcare). hRBP4 in 10 mM sodium acetate buffer (pH 4) was immobilized at a concentration of 20 μg/mL and a flow rate of 30 µL/min to a density of 500 resonance unit (RU) on flow cell 2. Flow cell 1 served as a reference surface. Single-cycle kinetic experiments were performed using increasing concentrations of binder in the absence or presence (5 µM) of A1120 in the running buffer (0.01 M Hepes, pH 7.4; 0.15 M NaCl; 3 mM ethylenediaminetetraacetic acid (EDTA); 0.005% vol/vol Surfactant P20; HBS-EP, GE Healthcare). Concentrations of RS3 and RS5 ranged from 6.25 to 100 nM. In additional experiments only conducted in the absence of A1120, concentrations of RS3 and RS5 ranged from 937.5 to 15,000 nM. Association times were 60 s, dissociation times were 60 s, and flow rate was set to 30 µL/min. To determine the equilibrium dissociation constant *K*_D_, equilibrium response units were plotted against analyte concentrations, and the data were fitted to a 1:1 binding model including a term for bulk refractive index contribution. For titration of A1120, hRBP4 was immobilized on a CM5 chip as described above and equilibrated with running buffer containing the respective A1120 concentration (ranging from 0 to 4,374 nM) for 30 min, followed by 5 min association time with 100 nM RS3 (flow rate of 30 µL/min) in the presence of the same A1120 concentration used during the 30 min equilibration phase. The steady-state level reached after those 5 min was used for calculation of the A1120-dependant EC_50_. After each measurement, the chip was regenerated with one injection of 4 M MgCl_2_ (60 s, 30 µL/min). Data analysis was performed with the Biacore T200 Evaluation Software (GE Healthcare), and steady-state levels, which were reached after 5 min association, were plotted against A1120 concentration.

### Differential Scanning Calorimetry.

DSC experiments were performed with the PEAQ Differential Scanning Calorimeter Automated (Malvern Panalytical); 80 µM of the respective binder in PBS were heated up from 20 °C to 110 °C with a heating rate of 1 °C/min. Data analysis was performed with the PEAQ-DSC analysis software (Malvern Panalytical). After buffer baseline subtraction and normalization for protein concentration, transitions were fitted with a non-two-state thermal unfolding model.

### Cytotoxicity Assay.

Analysis of the cytotoxic potential of CAR T cells was performed by cocultivating CAR T cells with luciferase expressing target cells at an effector:target (E:T) ratio of 2:1 in round-bottom 96-well plates for 4 h at 37 °C either in the presence or absence of 5 µM A1120 in RPMI GlutaMAX (Life Technologies) supplemented with 10% fetal calf serum (Sigma-Aldrich) and 100 U/mL penicillin and 100 µg/mL streptomycin (both Life Technologies). Target cells were either Jurkat cells electroporated with 3 µg mRNA encoding CD19 and/or 3 µg mRNA encoding luciferase or NALM6 cells as indicated. Target cells were incubated in 100 µL volume with 5 or 10% human serum (as indicated) for 10 min at 4 °C before effector CAR T cells were added. Following incubation, remaining living cells were quantified by addition of luciferin (150 µg/mL final concentration; Perkin-Elmer), and luciferase activity was measured after 20 min by using the EnSpire Multimode plate reader. The percentage of lysis was determined with the following formula (RLU stands for relative light units):

$$\mathrm{lysis}\left(\%\right)\mathrm{=100}-\frac{\left(\text{RLU from well with effector and target cell coculture}\right)}{\left(\text{RLU from well with target cells only}\right)}\times 100.$$

### Activation Assay Using Reporter Jurkat Cells.

The function of the ON-switch in a CAR was analyzed by determining the activation of the transcription factors NFAT and NFκB in reporter Jurkat cells. These reporter cells were generated by introducing an NFAT::eCFP reporter construct into a highly sensitive NF-κB::eGFP Jurkat cell line described previously (57). A highly sensitive cell line containing both reporters was established using a screening strategy described previously (58). Reporter Jurkat cells electroporated with 5 µg mRNA of the indicated CAR construct, and 3 µg of mAmetrine mRNA were cocultured with NALM6 target cells at an effector:target ratio of 1:2 in 96 round-bottom wells. NALM6 target cells were blocked with 10% human serum and 10% heat-inactivated human IgG for 10 min at 4 °C prior to the addition of effector cells. Where indicated, 1 µM of soluble hRBP4 (recombinantly produced in yeast as described in *SI Appendix*) was added at the beginning of the coculture. After 20 h of coincubation, expression of the fluorescent proteins e-CFP and e-GFP was assessed by flow cytometric analysis.

### Data Availability Statement.

Structural data for this study have been deposited in the Research Collaboratory for Structural Bioinformatics (RCSB) Protein Data Bank (PDB) (https://www.rcsb.org/) under accession number 6QBA (36). All other data discussed in the paper are available to readers upon request.

## Supplementary Material

Supplementary File
