# Supplementary material for: A conformation-specific ON-switch for controlling CAR T cells with an orally available drug
Source: Proc Natl Acad Sci U S A. 2020 Jun 17;117(26):14926–35. doi: 10.1073/pnas.1911154117 (PMC7334647; doi:10.1073/pnas.1911154117)
Supplement: Supplementary File [file pnas.1911154117.sapp.pdf]

Supplementary Information for

A conformation-specific ON-switch for controlling CAR T cells with an orally available drug

Charlotte U. Zajc[a,b], Markus Dobersberger[a], Irene Schaffner[c], Georg Mlynek[d], Dominic Pühringer[d], Benjamin Salzer[a,b], Kristina Djinović-Carugo[d,e], Peter Steinberger[f], Annika De Sousa Linhares[f], Nicole J. Yang[g], Christian Obinger[h], Wolfgang Holter[a,i], Michael W. Traxlmayr[b,h,1,2] and Manfred Lehner[a,b,i,1,2]

[a]St Anna Children's Cancer Research Institute, 1090 Vienna, Austria; [b]Christian Doppler Laboratory for Next Generation CAR T Cells; [c]BOKU Core Facility Biomolecular & Cellular Analysis, BOKU-University of Natural Resources and Life Sciences, 1190 Vienna, Austria; [d]Department of Structural and Computational Biology, Max Perutz Labs, University of Vienna, 1030 Vienna, Austria; [e]Department of Biochemistry, Faculty of Chemistry and Chemical Technology, University of Ljubljana, 1000 Ljubljana, Slovenia; [f]Institute of Immunology, Medical University of Vienna, 1090 Vienna, Austria; [g]Department of Immunology, Harvard Medical School, Boston, MA 02115; [h]Department of Chemistry, Institute of Biochemistry, BOKU-University of Natural Resources and Life Sciences, 1190 Vienna, Austria; [i]St. Anna Kinderspital, Department of Pediatrics, Medical University of Vienna, 1090 Vienna, Austria

[1]these authors contributed equally to this work: Michael W. Traxlmayr, Manfred Lehner
[2]To whom correspondence may be adressed: michael.traxlmayr@boku.ac.at or manfred.lehner@ccri.at

This PDF file includes:

Supplementary text (Materials & Methods)
Figures S1 to S7
Tables S1
Legends for Figures S1 to S7
Legends for Table S1

Supplementary text (Materials & Methods)

Screening for binders using yeast display

Yeast-displayed libraries based on rcSso7d (libraries rcSso7d-11 and rcSso7d-18, library size of 1.4×10^9 each(1)) and the library G4 based on the FN3 domain(2) with a library size of 2.5×10^8 were used. *S. cerevisiae* (strain EBY100) was grown in SD-CAA medium (20 g/L glucose, 6.7 g/L yeast nitrogen base, 5 g/L casamino acids, 11.85 g/L sodium citrate dihydrate and 7.4 g/L citric acid monohydrate) at 30°C overnight while shaking. Cells were passaged to an OD_{600} of 1, followed by incubation at 30°C while shaking. When an OD_{600} of 4 was reached, cells were centrifuged and resuspended to an OD_{600} of 1 in SG-CAA medium (2 g/L glucose, 20 g/L galactose, 6.7 g/L yeast nitrogen base, 5 g/L casamino acids, 10,2 g/L disodium hydrogen phosphate and 4,82 g/L sodium phosphate monobasic). Cells were incubated at 20°C overnight while shaking and subsequently harvested by centrifugation. Two cycles of bead selection were performed with magnetic streptavidin-coated Dynabeads (Life Technologies) loaded with biotinylated hRBP4 as described previously(2-4). To avoid the selection of non-specific binders or binders which are specific for streptavidin, negative selections (with bare beads) were performed between cycles of positive selection. In all bead selections, A1120 (Sigma-Aldrich) was present at a concentration of 5 µM. After the second bead-selection cycle, error prone PCR (epPCR) was conducted in order to introduce additional mutations into rcSso7d and FN3 genes. The template DNA was subjected to 19 PCR cycles using nucleotide analogs (2 µM of 8-oxo-2′-deoxyguanosine-5′-triphosphate, 8-oxo-dGTP, and 2µM 2′-deoxy-p-nucleoside-5′-triphosphate, dPTP) and the primers epSso_fwd (5′-

GGCTCTGGTGGAGGCGGTAGCGGAGGCGGAGGGTCGGCTAGC-3´) and epSso_rev (5´-CTATTACAAGTCCTCTTCAGAAATAAGCTTTTGTTCGGATCC-3´) for the rcSso7d-based binders and the primers FN3_fwd (5´-CGACGATTGAAGGTAGATACCCATACGACGTTCCAGACTACGCTCTGCAG-3´) and FN3_rev (5´-ATCTCGAGCTATTACAAGTCCTCTTCAGAAATAAGCTTTTGTTCGGATCC-3´) for the FN3-based binders, respectively. The resulting product was used as a template for further amplification by a second PCR using the same primers that were also used for epPCR. Afterwards, EBY100 cells were electroporated with linearized pCTCON2 vector(3, 5) and insert DNA (PCR product) as described previously(6). After a third round of bead-selection (comprising 3 negative and 1 positive selection), libraries were further enriched by fluorescence activated cell sorting (FACS). Staining of yeast cells was performed in PBS supplemented with 0.1% bovine serum albumin (BSA, Sigma-Aldrich). In positive selections (i.e. selection for hRBP4 binding) A1120 was added to a final concentration of 5 µM, whereas in negative selections (i.e. selection for non-binding to hRBP4) no A1120 was present. Cells were incubated with varying concentrations of biotinylated hRBP4 (depending on the selection round) and 5 µg/mL mouse anti-c-myc antibody (clone 9E10, Thermo Fisher Scientific) at 4°C for 1 h while shaking. After a washing step, a secondary labeling step was performed with 20 µg/mL streptavidin-Alexa Fluor 647 and 20 µg/mL anti-mouse IgG-Alexa Fluor 488 (both from Thermo Fisher Scientific) for 20 min at 4°C while shaking. After a final washing step, cells were sorted using a FACS Aria Fusion (BD). In some sorting rounds, cells were stained with non-biotinylated hRBP4 as primary reagent followed by secondary staining with 1 µg/mL anti-

HA-Alexa Fluor 647 (clone 16B12, BioLegend) and 5 µg/mL Penta-His-Alexa Fluor 488 (Qiagen, recognizing the His_6-tagged hRBP4), to avoid enrichment of binders recognizing biotinylated epitopes. Between the second and third FACS selection, another epPCR was performed. In total, 7 rounds of FACS were performed.

Expression and purification of hRBP4

Human RBP4 (residues 19-201) with an N-terminal His-Tag in the pPICZα A vector was expressed in *P. pastoris* strain KM71H. The yeast strain KM71H transformed with the hRBP4-construct(7) was kindly provided by John Findlay. Cells were cultivated in YPG (2% peptone, 1% yeast extract, 1% glycerol) supplemented with Zeocin (100 µg/mL) at 30°C and 180 rpm and induced at an OD_{600} of 2 by centrifugation and resuspension in YPM (2% peptone, 1% yeast extract, 1% methanol) and further incubation at 20°C and 180 rpm for 3 days with daily addition of methanol to a final concentration of 1%. The supernatant was harvested by 2-step centrifugation (1500 g, 15 min, 4°C and 12 200 g, 25 min, 4°C) and diafiltration was performed with the diafiltration TFF system (Merck Millipore) using Pellicon XL membranes (5 kDa cut-off, Merck Millipore) to exchange the medium with loading buffer (50 mM sodium phosphate, 500 mM NaCl and 5 mM imidazole, pH 7.4). The following purification protocol was adapted from Wysocka-Kapcinska et al.(7). Briefly, the diafiltrated supernatant was applied to a HisTrap FF column (GE healthcare) connected to an ÄKTA HPLC purifier system (GE healthcare). Elution was performed using a linear imidazole gradient (from 5 to 500 mM imidazole in a 50 mM sodium phosphate buffer, pH 7.4, containing 500 mM NaCl). Fractions containing hRBP4 were pooled and concentrated using Amicon Ultra-15 10K centrifugal filters (Merck Millipore).

Buffer was exchanged to phosphate buffered saline (PBS, pH 7.4). Before preparative size exclusion chromatography (SEC), a fraction of RBP4 was labelled with biotin using the EZ-Link Sulfo-NHS-LC-LC-Biotin kit (Thermofisher Scientifc). Subsequently, preparative SEC was performed using a Superdex 200 column (10 mm x 300 mm, GE Healthcare) to remove remaining unbound biotin molecules.

Soluble expression of selected hRBP4-binders

After the selection, 96 enriched clones were sequenced. 16 different hRBP4-binders (9 rcSso7d- and 7 FN3-based binders) were chosen based on their sequence and used for transformation of EBY100 cells. The affinity of single clones was determined by flow cytometry and titration of hRBP4. The 8 most promising binders (5 rcSso7d- and 3 FN3-based binders) were chosen for soluble expression. For this purpose, binders were sub-cloned into the pE-SUMO-vector (LifeSensors) and expressed as fusion proteins with His_6-tagged small ubiquitin-like modifier (SUMO) protein. After transformation of Rosetta (DE3) *E. coli* cells with the sequence-verified plasmids, cultures were incubated in LB medium supplemented with kanamycin (50 µg/mL) and chloramphenicol (34 µg/mL) at 37°C overnight while shaking. Cells were diluted in terrific broth (12 g/L tryptone, 24 g/L yeast extract, 4% glycerol, 2.31 g/L KH_2PO_4 and 16.43 g/L K_2HPO_4*$3H_2O$) supplemented with antibiotics to an OD_{600} of 0.4 and further incubated at 37°C. Expression was induced at an OD_{600} of 2 by addition of 1 mM isopropyl β-D-1-thiogalactopyranoside (IPTG) and cells were further cultured overnight at 20°C. Cells were harvested by centrifugation (5000 g, 20 min, 4°C) and resuspended in sonication buffer (50 mM sodium phosphate, 300 mM NaCl, 3% glycerol, 1% Triton X-100, pH 8). After sonication and centrifugation (20 000

g, 30 min, 4°C), purification of the supernatants containing the His_6-SUMO-fusion proteins was performed using the TALON metal affinity resin (Clontech). Supernatants were supplemented with 10 mM imidazole to prevent unspecific binding and were applied to the column twice, followed by two washing steps with equilibration buffer (50 mM sodium phosphate, 300 mM NaCl, pH 8) containing increasing concentrations of imidazole (5-15 mM). Elution was performed with equilibration buffer supplemented with 250 mM imidazole. The buffer was exchanged to PBS using Amicon Ultra-15 10K Centrifugal filters (Merck Millipore). The fusion proteins were digested overnight at room temperature with SUMO-protease 1 (1 mg enzyme per 100 mg fusion protein), resulting in cleavage of the hexahistidine tag and SUMO protein from the respective binder. The digested protein was again purified with TALON metal affinity resin. Since the binder proteins did not contain any hexahistidine tag after SUMO-digestion, they were collected from the flow-through. Finally, the buffer was exchanged with PBS using Amicon Ultra-15 3K centrifugal filters (Merck Millipore).

Size exclusion chromatography

For analytical SEC analysis, the respective binder in running buffer (PBS supplemented with 200 mM NaCl) was filtered through a 0.1 µm Ultrafree-MC filter (Merck Millipore). Subsequently, 25 µg protein were applied to a Superdex 200 column (10 mm x 300 mm, GE Healthcare) connected to an HPLC Prominence LC20 system (Shimadzu) and eluted with a flow rate of 0.75 mL/min at 25°C.

Expression and purification of hRBP4 with cleavable His_6-Tag for crystallization

For crystallization, hRBP4 was cloned into the pPICZα A vector with an additional cleavage site after the His_6-tag for recognition by the human rhinovirus 3C (HRV 3C) protease in order to produce protein without a tag. The *P.pastoris* strain X-33 was transformed with the sequence-verified plasmid and the protein was expressed and purified as described above. After purification on a His Trap column (GE Healthcare), the protein was incubated with HRV 3C protease (fused to a glutathione-S-transferase tag) for cleavage of the His_6 tag (digestion overnight at 4°C with ten-fold excess of HRV 3C protease). Finally, the digested sample was loaded onto a GSTrap HP column (GE Healthcare) to retain the protease and onto a His Trap column (to remove the cleaved tag), followed by a final purification by preparative SEC using a Superdex 200 column (10 mm x 300 mm, GE Healthcare).

Crystallization of the hRBP4-A1120-RS3 complex

Purified hRBP4 in PBS was mixed with 1.5-fold molar excess of RS3 in PBS and 500 µM A1120 and concentrated to a total protein concentration of 21 mg/mL using Amicon Ultra centrifugal filter units (Merck Millipore). Crystallization was performed using the sitting drop vapor diffusion method in SWISSCI MRC three-well crystallization plates (Molecular Dimensions). Crystallization plates with commercially available screens were set up using the mosquito crystallization robot (TTP LabTech). The reservoir was filled with 40 µL of precipitant solution and different ratios of protein to precipitant (150:200 nL, 200:200 nL and 250:200 nL) were applied. Crystallization plates were stored in a Formulatrix RI-1000 imaging device at 22°C. Crystals were obtained with the JCSG-plus

screen (Molecular dimensions) in the following condition: 0.2 M Zn acetate, 0.1 M imidazole pH 8 and 20% PEG 3K. Crystals were soaked with mother liquor supplemented with 20% 2-methyl-2,4-pentanediol (MPD), collected with cryo-loops and flash-vitrified with liquid nitrogen.

Data collection and structure refinement

Datasets were collected at beamline ID30A-3 (8) of European Synchrotron Radiation Facility (ESRF, Grenoble, France) at 100 K using a DECTRIS Eiger X 4M detector. The dataset was processed with XDS and symmetry equivalent reflections merged with XDSCONV (9). Intensities were not converted to amplitudes. Initially we used a conservative high-resolution cutoff 1.91 Å (CC1/2=67.5; I/SIGMA=1.57)(10). The phase problem was solved by molecular replacement using phenix.phaser taken the search models 4O9S and chain A of 1XYI. The model was further improved by iterative cycles of manual model building using COOT (11) and maximum likelihood refinement using phenix.refine (12). Phenix.refine converted intensities into amplitudes using the French and Wilson algorithm (13). The final high-resolution cutoff was based on performing paired refinement using the PDB_REDO webserver (14). Final stages of refinement included Translation Liberation Screw (TLS) parameters, isotropic B-factor model, automated addition of hydrogens and water molecules, optimization of X-ray/ADP weight, and optimization of X-ray/stereochemistry weight. The model was validated with MolProbity(15). Figures were prepared with PyMOL Molecular Graphics System (Version

1.3, Schrödinger, LLC). Atomic coordinates have been deposited in the Protein Data Bank under the accession code 6QBA.

Cell culture

Primary $CD3^+$ T cells were isolated through de-identified leukapheresis of voluntary healthy donors using the RosetteSep Human T cell enrichment cocktail (Stem cell technologies) according to the manufacturer´s instructions. T cells were activated with Human T-Activator CD3/CD28 Dynabeads (Life Technologies) and cultured in RPMI GlutaMAX (Life Technologies) supplemented with 10% fetal calf serum (FCS, Sigma-Aldrich), 100 U/mL penicillin, 100 µg/mL streptomycin (both Life Technologies) and 200 U/mL IL-2 (Peprotech) at 37°C. Activated T cells were split every second day and cultured at densities between 0.3 to 1×10^6 cells/mL. The reporter Jurkat cell line for analyzing the activity of the transcription factors NFAT (e-CFP) and NFκB (e-GFP) and NALM6 cells were cultured in the same medium as primary T cells but without additional IL-2. For the cytotoxicity assay NALM6 target cells were transduced with a lentiviral vector encoding Luciferase and GFP. For that purpose, production of supernatant of pantropic VSV.G pseudotyped lentivirus was performed from Lenti-X 293T cells (Clontech Laboratories) with a third-generation Puromycin-selectable pCDH transgene expression vector (pCDH-EF1-MCS-T2A-Puro cDNA vector, System Biosciences) and second-generation viral packaging plasmids pMD2.G and psPAX2 (both obtained from Addgene, plasmids #12259 and #12260, respectively). Co-transfections were performed using Purefection Transfection Reagent (System Biosciences) according to the manufacturer´s instructions. Supernatants were collected 36 and 60 h after transfection and concentrated using the

Lenti-X concentrator (Clontech Laboratories). For the transduction of NALM6 cells, 20 000 cells were seeded in 96 wells and lentiviral supernatant containing the vector encoding for Luciferase was added in different dilutions (1:4, 1:20, 1:100). After 24 h, medium was exchanged and the cells were further cultured for 72 h at 37°C. Subsequent selection with puromycin (Sigma-Aldrich) was performed with concentrations between 0.5 and 8 µg/mL. After additional 48 h of cultivation, the transduction efficacy was determined by flow cytometric detection of GFP co-expressed by the vector and the transduced NALM6 cells were used to create a working cell bank.

Design of CARs and transfection of primary T cells and Jurkat cells

For construction of the ON-switch CAR chain I, the hRBP4-binder RS3 was incorporated into a second-generation CAR signaling backbone, comprising an IgG1-Fc spacer fused to a CD28 transmembrane and costimulatory domain and a CD3zeta signaling domain, respectively. The secreted chain II contained an FMC63-based scFv directed against human CD19 fused to IgG1-Fc via a $(G_4S)_2$ linker. The CAR chains were constructed by Gibson Assembly (NEB), using 0.02 - 0.5 pmol of PCR-amplified DNA fragments for assembly. The resulting constructs were amplified by PCR and subsequently used for *in vitro* transcription with the mMessage mMachine T7 Ultra Kit (Ambion) according to the manufacturer´s instructions. Resulting mRNAs were column purified with an adopted protocol using the RNeasy Kit (Qiagen). Briefly, RLT buffer from the kit and 1% β-mercaptoethanol were added. After addition of absolute ethanol, the mixture was loaded onto an RNeasy column and the purification was continued according to the manufacturer´s protocol. Purified mRNA was stored frozen at -80°C. Primary T cells were electroporated

with 5 µg mRNA using the square wave protocol (500 V, 5 ms, 4 mm cuvettes). Jurkat target cells were electroporated (square wave, 500 V, 3 ms, 4 mm cuvettes) with 3 µg mRNA encoding CD19 and/or 3 µg mRNA encoding luciferase. Jurkat reporter cells were electroporated with 5 µg mRNA of the respective CAR construct and 3 µg mRNA encoding the fluorescent protein mAmetrine (obtained from Addgene, plasmid #54505). All electroporations were performed with the Gene Pulser (Biorad). After electroporation, cells were incubated for 20 h at 37°C before they were used for further experiments.

Flow cytometric analysis of CAR expression

Cells were counted using Accucheck counting beads (Life Technologies) and propidium iodide for exclusion of dead cells. Washing and staining of cells was performed with ice cold buffer consisting of PBS (Life Technologies) supplemented with 0.2% human albumin (CSL Behring) and 0.02% sodium azide (Merck KGaA). Expression of CAR chain I was detected by blocking 5×10^4 cells with 10% (w/v) murine IgG1-κ (clone MOPC21, Sigma-Aldrich) for 10 min at 4°C followed by primary staining with biotinylated anti-human-IgG1-Fc-antibody (clone JDC-10, Southern Biotec) as primary antibody and phycoerythrin (PE)-conjugated streptavidin (eBioscience) as secondary staining reagent. Binding of chain II to chain I, which was co-expressed, was detected by staining 5×10^4 T cells with anti-penta-His-Alexa Fluor 488 antibody (Qiagen) for 30 min at 4°C. During the staining incubation and the subsequent washing step, 5 µM A1120 was present in the staining buffer to prevent dissociation of chain II.

Cytokine release by CAR T cells

The supernatants from the cytotoxicity assays described in the "Materials and Methods" section of the main manuscript were harvested after 4 h, followed by centrifugation (1600 rpm, 7 min) to remove remaining cells and stored at -20°C. For analysis of secreted cytokines, ELISA was performed using the Human IFN-γ ELISA Ready-SET-Go! kit and IL-2 Human ELISA Kit (bot kits from Thermofisher Scientific) according to the manufacturer´s instructions. Measurements were conducted using the EnSpire Multimode plate reader.

Fig. S1.

A

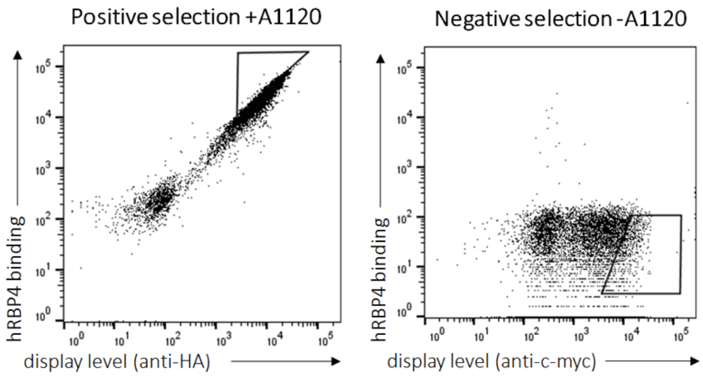

B

```
     1        10        20        30        40        50        60
RS1  ATVKFTYQGEEEQVDISKIKKVARYGQNIYFSYDEGGGAWDYGGVSEKDAPKELLQMLEKQ
RS2  ATVKLTYQGEEKQVDISKIKRVARYGQGIYFDYGEGGGAWGYGSVSEKDAPKELLQMLEKQ
RS3  ATVKLTYQGEEKQVDISKIKRVARYGQNIYFSYDEGGGAYDYGAVSEKDAPKELLQMLEKQ
RS4  ATAKFTYQGEEKQVDISKIKRVARYGQGIYFSYDEGGGAYGYGSVSEKDAPKELLQMLEKQ
RS5  ATVKFTYQGEEKQVDISKIKRVARYGQGIYFDYGEGGGAWGYGSVSEEDAPKELLQMLEKQ

     1        10        20        30        40        50        60
RF1  VSDVPRDLEVVAATPTSLLISWYYPNASHAGYYRVTYGETGGNSPVQEFTVPFSIRYTIAT
RF2  VSDVPRDLEVVAATPTSLLISWYYPNASHAGYYRITYGETGGNSPVQEFTVPFSIRYTIAT
RF3  VSDVPRDLEVVAATPTSLLISWYYPNASHAGYYRITYGETGGNSPVQEFTVPFSIRYTIAT
              70        80        90        100
RF1  ISGLKPGVDHTITVYAVTDYAYYYRSSEPISINYRTEIDKPSQ
RF2  ISGLKPGVDYTITVYAVTDYAYYYRLSEPISINYRTEIDKPSQ
RF3  ISGLKPGVDYTITVYAVTASCQYC--SYPISINYRTEIDKPSQ
```

C

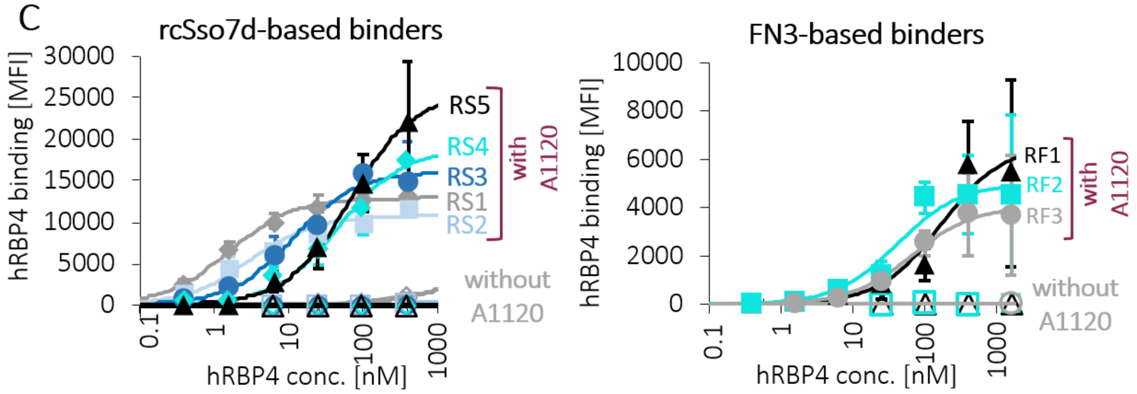

D

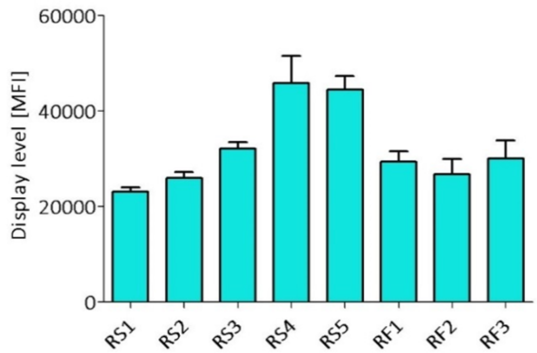

E

rcSso7d-based or FN3-based hRBP4 binder		Yeast display K_D (nM; mean ± S.D.) + A1120	-A1120
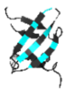	RS1	2 ± 0	n.a.*
	RS2	3 ± 0	n.a.*
	RS3	13 ± 7	n.a.*
	RS4	48 ± 6	n.a.*
	RS5	80 ± 50	n.a.*
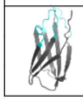	RF1	128 ± 75	n.a.*
	RF2	65 ± 42	n.a.*
	RF3	45 ± 14	n.a.*

Suppl. Fig. 1: (A) Gating strategy for the selection of yeast-displayed rcSso7d-based binders for specific interaction with A1120-loaded hRBP4. Representative flow cytometric data of 1 positive selection (in the presence of 5 µM A1120 and 100 nM hRBP4; dot plot on the left) and 1 negative selection (in the absence of A1120 and presence of 1000 nM hRBP4; dot plot on the right) are shown. **(B)** Amino acid sequences of enriched hRBP4-binders. Positions highlighted in cyan indicate the randomized positions within the respective library, dark blue positions show mutations which are different from the consensus sequence and positions colored in pink refer to mutations outside of the initially randomized binding site, which were potentially acquired during epPCR. **(C)** A1120-dependent binding of hRBP4 to enriched rcSso7d-based (termed RS) and FN3-based (termed RF) binders. Binders were displayed on the surface of yeast and analyzed in the presence of different hRBP4 concentrations. Shown are average MFIs ± S.D.s of 3 independent experiments. Filled and open symbols represent binding curves in the presence and absence of A1120, respectively. Data were fitted to a 1:1 binding model (solid lines) to calculate the K_D values shown in (E). **(D)** Display levels of hRBP4-binders shown in (C) on the surface of yeast. Shown are average MFIs ± S.D.s of displaying cells of 3 independent experiments. **(E)** Calculated K_D values of RS and RF binders shown in (C) as determined by titration on the surface of yeast. (*n.a.; not analyzable).

Fig. S2.

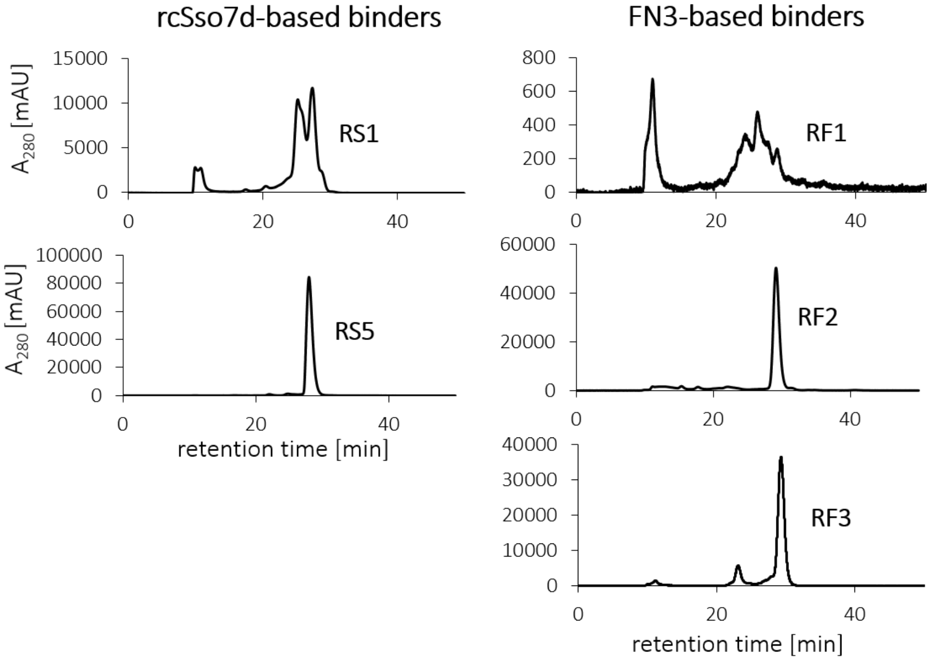

Supplemental Fig. 2: Size exclusion chromatography (SEC) profiles of selected rcSso7d- or FN3-based hRBP4-binders. One representative measurement of 3 independent experiments is shown.

Fig. S3.

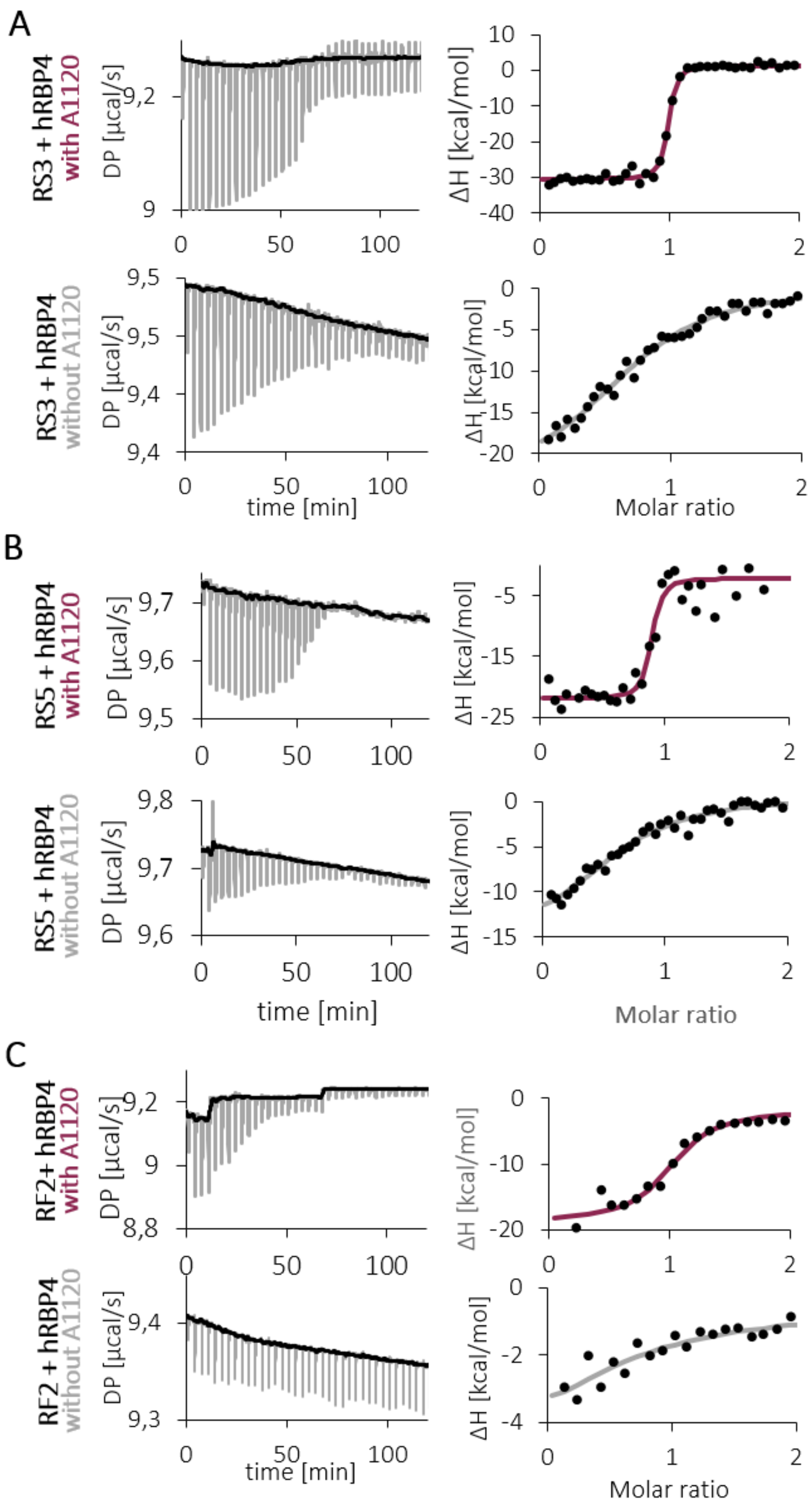

A
RS3 + hRBP4
with A1120
DP [µcal/s]
ΔH [kcal/mol]
RS3 + hRBP4
without A1120
time [min]
Molar ratio
B
RS5 + hRBP4
with A1120
RS5 + hRBP4
without A1120
C
RF2+ hRBP4
with A1120
RF2 + hRBP4
without A1120

Supplemental Fig. 3: **(A)** Analysis of affinity of one rcSso7d-based binder (RS3) to hRBP4 in the presence (50 µM) and absence of A1120 as determined by isothermal titration calorimetry (ITC). The integrated data were fitted to a one-set-of-sites binding model to calculate the K_D values shown in Figure 2E. **(B and C)** Binding of RS5 (B) and RF2 (C) to hRBP4 in the presence (50 µM) and absence of A1120 as determined by ITC measurements. The integrated data were fitted to one-set-of-sites binding model to calculate the K_D values in Fig. 2E. In (A), (B) and (C) one representative of four independent experiments is shown.

Fig. S4.

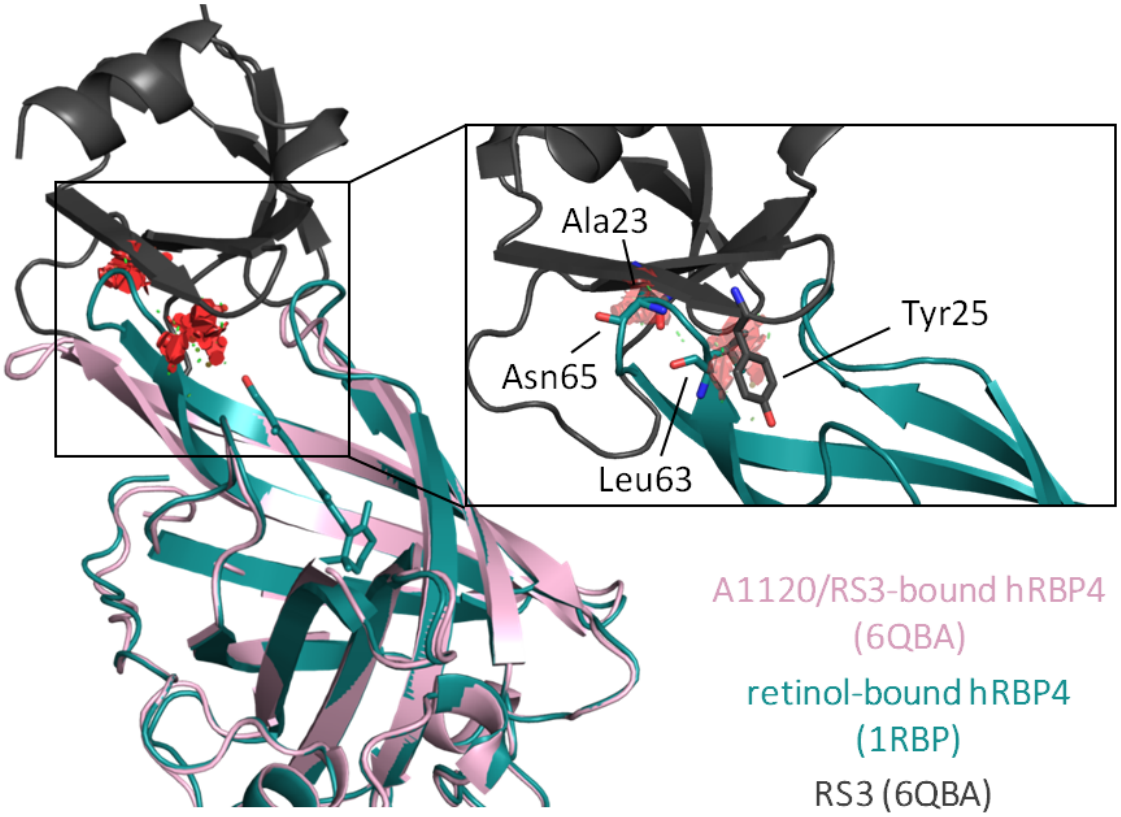

Supplemental Fig. 4: Sterical clashing of RS3 (dark grey) with retinol-bound hRBP4 (dark green, PDB ID code: 1RBP). The A1120-bound hRBP4 structure is represented in pink (PDB ID: 6QBA). The close-up view exemplarily shows the sterical clash of amino acids of retinol-bound hRBP4 (Asn65, Leu63) with RS3 (Ala23, Tyr25). All figures were generated using PyMOL Molecular Graphics System (Version 1.3, Schrödinger, LLC).

Fig. S5.

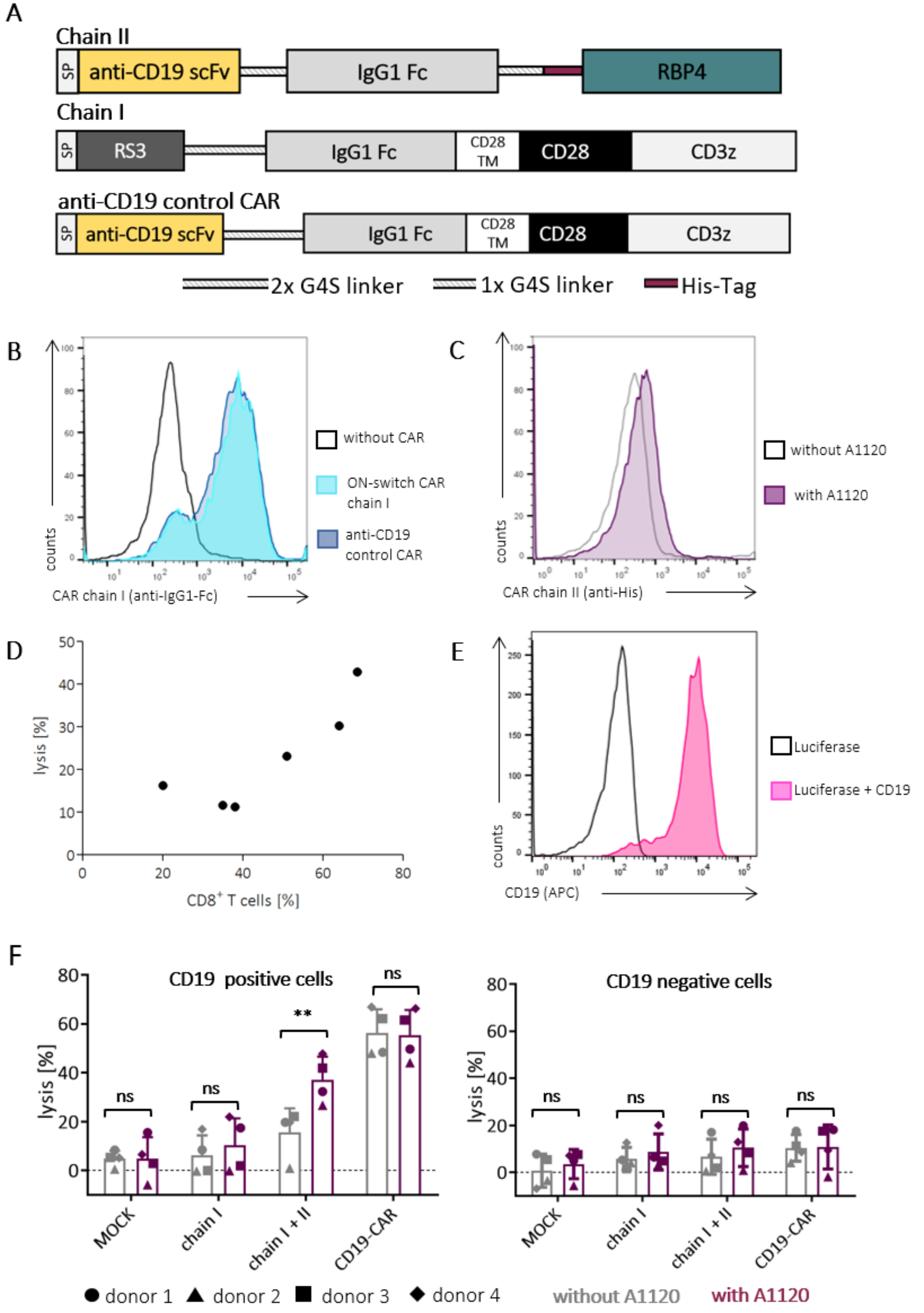
A
Chain II
SP
anti-CD19 scFv
IgG1 Fc
RBP4
Chain I
SP
RS3
IgG1 Fc
CD28 TM
CD28
CD3z
anti-CD19 control CAR
SP
anti-CD19 scFv
IgG1 Fc
CD28 TM
CD28
CD3z
2x G4S linker
1x G4S linker
His-Tag
B
counts
CAR chain I (anti-IgG1-Fc)
without CAR
ON-switch CAR chain I
anti-CD19 control CAR
C
counts
CAR chain II (anti-His)
without A1120
with A1120
D
lysis [%]
CD8+ T cells [%]
E
counts
CD19 (APC)
Luciferase
Luciferase + CD19
F
CD19 positive cells
lysis [%]
ns
**
MOCK
chain I
chain I + II
CD19-CAR
CD19 negative cells
donor 1
donor 2
donor 3
donor 4
without A1120
with A1120

Supplemental Fig. 5: **(A)** Representation of the components of the ON-switch CAR constructs and the anti-CD19 control CAR. Signal peptides were derived from either CD33 (ON-switch CAR chain I and the anti-CD19 control CAR) or hRBP4 (ON-switch CAR chain II). TM= transmembrane domain. **(B)** CAR expression in primary human T cells used in a cytotoxicity assay as determined by flow cytometry. **(C)** Staining of chain II bound to chain I expressed in primary T cells. **(D)** Correlation between the percentage of $CD8^{+}$ T cells and target cell lysis in the negative control (i.e. primary human T cells transfected without any mRNA) of the cytotoxicity assay shown in Fig. 5b. **(E)** Expression of CD19 in Jurkat cells after electroporation with either only luciferase mRNA or CD19 and luciferase mRNA. The stainings shown in b, c and e were performed 20 h after electroporation. One representative staining out of a minimum of 4 independent experiments is shown. **(F)** Primary human T cells were electroporated with mRNA encoding either chain I only, or both chain I and II of the ON-switch CAR, or an anti-CD19 control CAR. T cells electroporated without mRNA ("MOCK") were included as a negative control. Lysis of Jurkat target cells (electroporated with CD19 mRNA and/or luciferase mRNA) in the presence (5 µM) or absence of A1120 was determined after 4 h of co-culture with CAR T cells (E:T = 2:1). Target cells were blocked with 10% human serum and 10% human IgG for 15 min at 4°C before effector cells were added. Data from 4 independent experiments with primary T cells from 4 different donors are shown. Statistical significance was calculated with GraphPad using the paired two-tailed Student´s t test. (*** = $p<0.001$, ** = $p<0.01$, * = $p<0.05$).

Fig. S6.

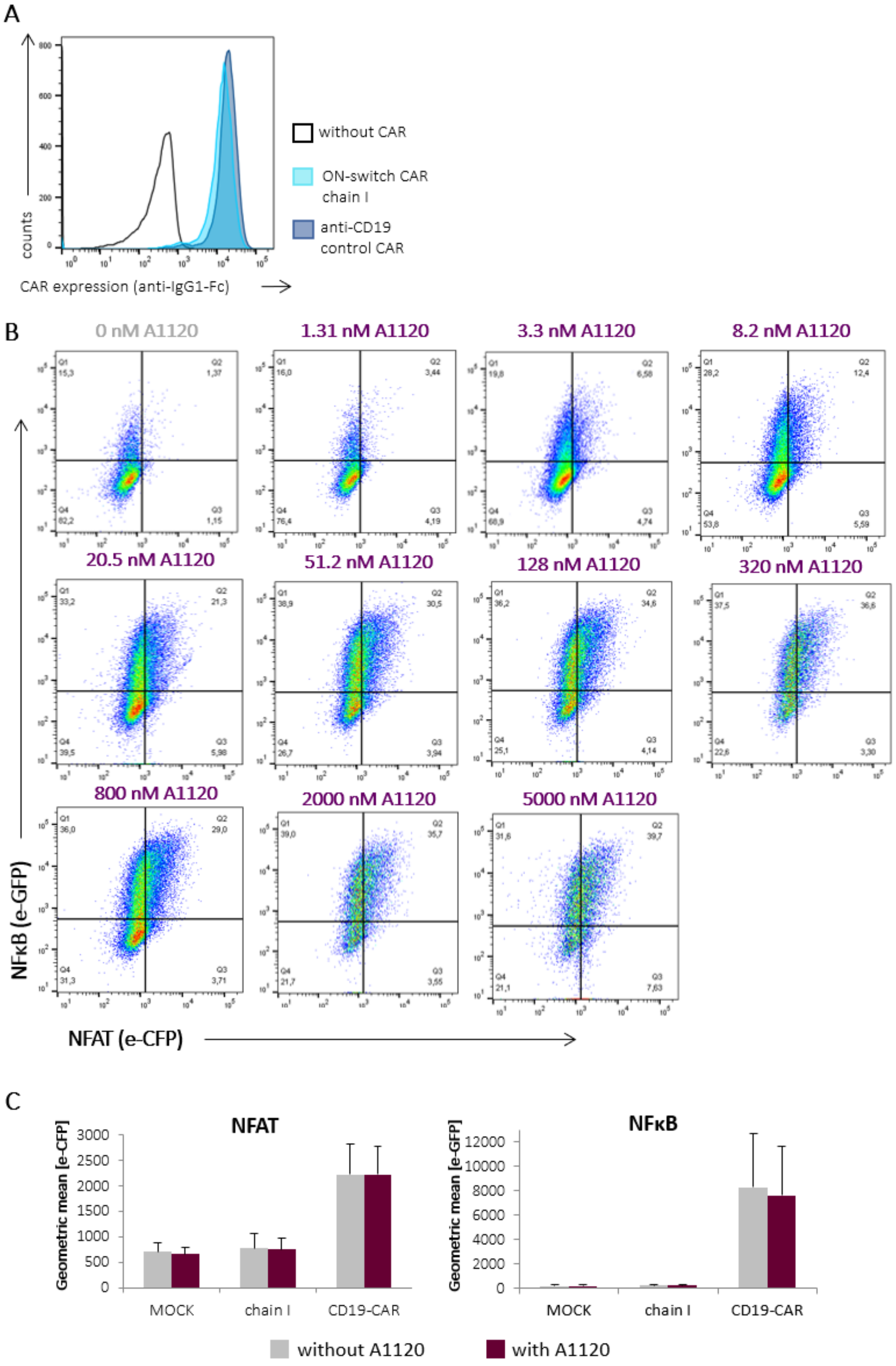
A
without CAR
ON-switch CAR chain I
anti-CD19 control CAR
counts
CAR expression (anti-IgG1-Fc)
B
0 nM A1120
1.31 nM A1120
3.3 nM A1120
8.2 nM A1120
20.5 nM A1120
51.2 nM A1120
128 nM A1120
320 nM A1120
800 nM A1120
2000 nM A1120
5000 nM A1120
NFκB (e-GFP)
NFAT (e-CFP)
C
NFAT
NFκB
Geometric mean [e-CFP]
Geometric mean [e-GFP]
MOCK
chain I
CD19-CAR
without A1120
with A1120

Supplemental Fig. 6: (A) Expression of the anti-CD19 control CAR and chain I of the ON-switch CAR in reporter Jurkat cells as determined by flow cytometry. **(B)** Expression of e-CFP and e-GFP in reporter Jurkat cells electroporated with chain I and II of the ON-switch CAR and co-cultured with NALM6 target cells (E:T 1:2) in the presence of different concentrations of A1120. Expression was analyzed by flow cytometry after 20 h of co-culture. **(C)** Expression of e-CFP and e-GFP in reporter Jurkat cells electroporated without mRNA (“MOCK”), or mRNA encoding for either chain I or the anti-CD19 control CAR after 20 h of co-culture with NALM6 target cells in the absence or presence of 5 μM A1120. Analysis was performed with FlowJo.

Fig. S7.

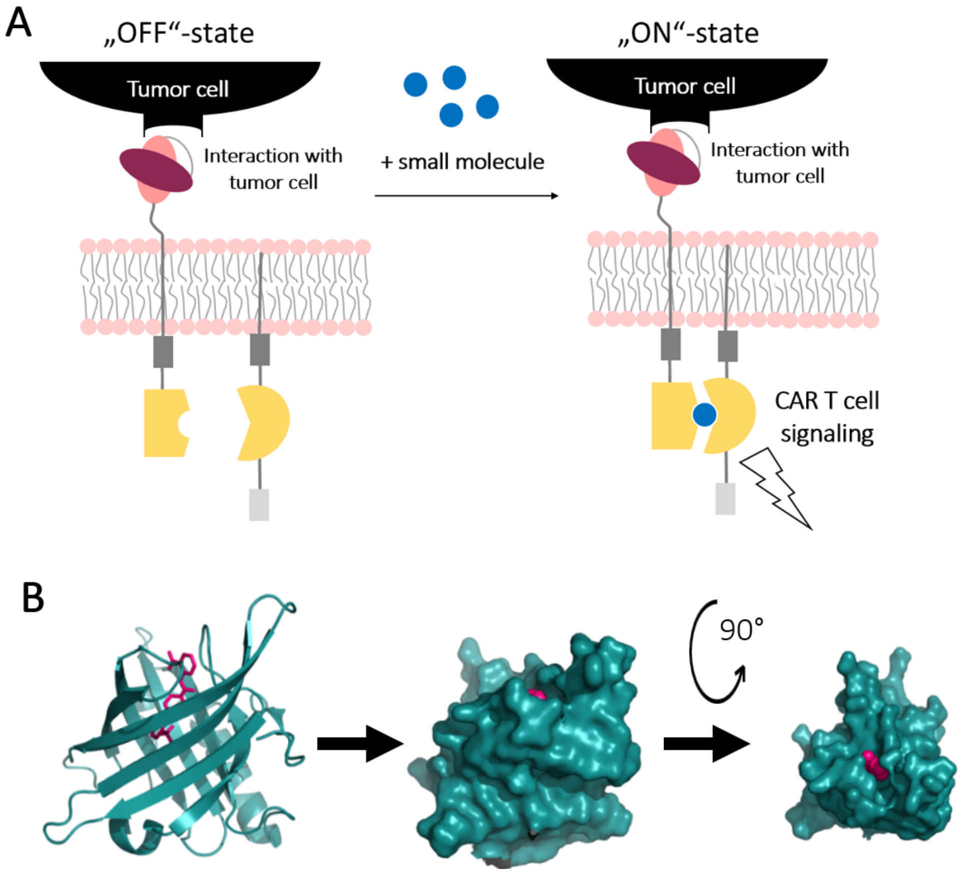

Supplemental Fig. 7: (A) Schematic representation of the ON-switch CAR system used by Lim and colleagues based on the FRB/FKBP-protein domains and a rapalog. **(B)** Crystal structure of hRBP4 bound to A1120 (PDB: 3FMZ) in the cartoon and surface representation. The two representations on the left and in the middle are in the same orientation, whereas the one on the right is rotated by 90°. The figures were generated using PyMOL Molecular Graphics System (Version 1.3, Schrödinger, LLC).

Table S1.

Table 1. Data collection and refinement statistics.

PDB ID	**6QBA**
Wavelength	0.968
Resolution range	40.25 - 1.8 (1.864 - 1.8)
Space group	P 21 21 21
Unit cell	44.456 77.614 80.509 90 90 90
Total reflections	177411 (18253)
Unique reflections	26332 (2596)
Multiplicity	6.7 (7.0)
Completeness (%)	99.27 (99.46)
Mean I/sigma(I)	9.65 (0.75)
Wilson B-factor	28.60
R-merge	0.1395 (2.098)
R-meas	0.1512 (2.263)
R-pim	0.05767 (0.843)
CC1/2	0.998 (0.26)
CC*	0.999 (0.642)
Reflections used in refinement	26332 (2595)
Reflections used for R-free	1679 (164)
R-work	0.1881 (0.3296)
R-free	0.2207 (0.3222)
CC (work)	0.959 (0.595)
CC (free)	0.957 (0.639)
Number of non-hydrogen atoms	2182
macromolecules	1906
ligands	67
solvent	209
Protein residues	244
RMS (bonds)	0.007
RMS (angles)	0.88
Ramachandran favoured (%)	97.42
Ramachandran allowed (%)	21217
Ramachandran outliers (%)	0.00
Rotamer outliers (%)	0.00
Clashscore	43437
Average B-factor	36.65
macromolecules	35.54
ligands	45.30
solvent	44.08
Number of TLS groups	18

[a] Values in parentheses represent the highest resolution shell.

Supplemental Table 1: Data collection and refinement statistics for the hRBP4-A1120-RS3 complex depicted in Fig. 4a. (PDB ID code: 6QBA).

References

1. M. W. Traxlmayr *et al.*, Strong Enrichment of Aromatic Residues in Binding Sites from a Charge-neutralized Hyperthermostable Sso7d Scaffold Library. *The Journal of biological chemistry* **291**, 22496-22508 (2016).
2. B. J. Hackel, M. E. Ackerman, S. W. Howland, K. D. Wittrup, Stability and CDR composition biases enrich binder functionality landscapes. *J Mol Biol* **401**, 84-96 (2010).
3. A. Angelini *et al.*, Protein Engineering and Selection Using Yeast Surface Display. *Methods Mol Biol* **1319**, 3-36 (2015).
4. M. Ackermann *et al.*, Highly Avid Magnetic Bead Capture: An Efficient Selection Method for de novo Protein Engineering Utilizing Yeast Surface Display. *Biotechnology Progress* **25**, 773-783 (2009).
5. G. Chao *et al.*, Isolating and engineering human antibodies using yeast surface display. *Nat Protoc* **1**, 755-768 (2006).
6. T. F. Chen, S. de Picciotto, B. J. Hackel, K. D. Wittrup, Engineering fibronectin-based binding proteins by yeast surface display. *Methods Enzymol* **523**, 303-326 (2013).
7. M. Wysocka-Kapcinska, J. A. Campos-Sandoval, A. Pal, J. B. Findlay, Expression and characterization of recombinant human retinol-binding protein in Pichia pastoris. *Protein Expr Purif* **71**, 28-32 (2010).
8. P. Theveneau *et al.*, The Upgrade Programme for the Structural Biology beamlines at the European Synchrotron Radiation Facility – High throughput sample evaluation and automation. *Journal of Physics: Conference Series* **425**, 012001 (2013).
9. W. Kabsch, Xds. *Acta crystallographica. Section D, Biological crystallography* **66**, 125-132 (2010).
10. P. A. Karplus, K. Diederichs, Linking crystallographic model and data quality. *Science* **336**, 1030-1033 (2012).
11. P. Emsley, B. Lohkamp, W. G. Scott, K. Cowtan, Features and development of Coot. *Acta crystallographica. Section D, Biological crystallography* **66**, 486-501 (2010).
12. Y. W. Lin, The broad diversity of heme-protein cross-links: An overview. *Biochim Biophys Acta* **1854**, 844-859 (2015).
13. S. French, K. Wilson, On the treatment of negative intensity observations. *Acta crystallographica. Section D, Biological crystallography*, 517-528 (1978).
14. R. P. Joosten *et al.*, PDB_REDO: automated re-refinement of X-ray structure models in the PDB. *Journal of applied crystallography* **42**, 376-384 (2009).
15. I. W. Davis, L. W. Murray, J. S. Richardson, D. C. Richardson, MOLPROBITY: structure validation and all-atom contact analysis for nucleic acids and their complexes. *Nucleic acids research* **32**, W615-619 (2004).
